# Research on an evaluation rubric for promoting user’s continuous usage intention: a case study of serious games for Chinese cultural heritage

**DOI:** 10.3389/fpsyg.2024.1300686

**Published:** 2024-02-15

**Authors:** Pingting Mao, Dong Min Cho

**Affiliations:** ^1^Department of Design and Manufacturing Engineering, Jeonbuk National University, Jeonju-si, Republic of Korea; ^2^Department of Industrial Design, Jeonbuk National University, Jeonju, Republic of Korea

**Keywords:** serious games, cultural heritage, continuous usage intention, evaluation rubric, Delphi method, analytic hierarchy process, fuzzy comprehensive evaluation method

## Abstract

The sustainable development of serious games dedicated to Chinese cultural heritage faces challenges due to the lack of prolonged user engagement and iterative optimization based on long-term user feedback. This situation not only hinders the sustainable growth of these games but also limits the transmission of Chinese cultural heritage, a problem that demands urgent attention yet remains under-acknowledged. This study synthesizes literature to unearth user needs from three dimensions: motivational use, quality requirements of games, and continuous usage intention. It identifies 14 influential factors, including cognitive satisfaction, immersion satisfaction, and achievement satisfaction. Drawing from the User Experience Rubric for Educational Games-CH (UEREG-CH) evaluation rubric and the Delphi method, these factors are expanded into an evaluation system model comprising six primary indicators, 14 secondary indicators, and 38 tertiary indicators. The analytic hierarchy process (AHP) is utilized for expert scoring to assign weights to each indicator. This is combined with the fuzzy comprehensive evaluation method to arrive at a final user score of Z = 3.4228, indicating that the evaluation rubric is close to ‘good’ and has received positive user feedback. Ultimately, this generates an evaluation rubric tailored to the specific context of serious games for cultural heritage. By integrating qualitative and quantitative methods, this paper confirms the scientific and rational nature of the evaluation rubric. The study aims to establish a user-approved rubric that encourages continuous usage intention, thereby providing effective guidance for game developers and assisting users in selecting appropriate games, while also addressing the theoretical gap in the field of evaluation for serious games related to cultural heritage.

## 1 Introduction

**Figure 1:**
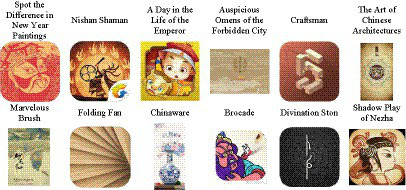
Serious games for Chinese cultural heritage.

This research focuses on Serious Games for Cultural Heritage, which are games developed through the integration of cultural heritage and serious gaming. The primary purpose of these games is to protect and disseminate a specific theme of cultural heritage, primarily through digital interactive formats. They often simulate and emulate traditional cultural skills. In this study, they are abbreviated as CH-SGs (Zhou and Xu, 2017). The interest in the cultural heritage domain within serious games arises from the current social context, where protecting and inheriting cultural heritage is a crucial historical mission for the national defense of independence and sovereignty. Many Chinese cultural heritage projects have been lost due to historical reasons and the complexity of their forms. Therefore, there is an urgent need to broaden the channels for disseminating cultural heritage and to find carriers for its living inheritance and innovation (Teng, 2019). CH-SGs, with their unique functional attributes, make cultural heritage knowledge and skills more accessible to users through digital network technologies and entertaining gaming methods, thereby stimulating their autonomous learning (Qin, 2017). Thus, CH-SGs play an essential role in preserving the authenticity of cultural heritage, enriching its presentation forms, lowering the barriers for user engagement, and assuming the crucial responsibility for the transformative development of cultural heritage. Hence, this paper emphasizes the importance of optimizing and improving CH-SGs for significant social relevance (Cai, 2020). Although CH-SGs possess positive social value and have become a new medium for presenting Chinese traditional culture to the world, supported strongly by the state, their practical development is not optimistic. Their development and usage in the Chinese gaming market are relatively scarce (Chen and Liu, 2019). To date, only about twenty games have been developed by renowned companies like Tencent Games and the Palace Museum (Figure 1). These games only offer a “one-time” experience of cultural heritage, sparking only brief interest in users and failing to elicit long-term usage feedback and real, objective evaluation standards to effectively improve game quality (Li, 2023). Likewise, theoretical research on these games is also scarce. In previous literature, the Chinese scholar Xu Haifeng focused on the Shenyang Imperial Palace’s material culture as a theme, emphasizing the game’s functional and aesthetic attributes to enhance user experience (Xu H. F., 2019). Li Haishi, through an analysis of games for intangible cultural heritage, summarized three design transformation paths, emphasizing the design expression of intangible cultural heritage, its visual transformation, and experience design (Li, 2019). Yang Xiao took Shaman culture as a starting point to analyze the factors influencing cognition to behavior in serious games, proving that games play a more effective role in the dissemination of intangible heritage (Yang, 2020). Previous literature has focused on CH-SGs’ cultural themes, game element design, and dissemination impact, but lacks consideration on how to promote continuous usage intention and exploration of user evaluation of such games.

The current evaluation theories for serious games generally adopt Gardner and Hatch, (1989) and Krathwohl et al. (1956). These evaluations focus on curriculum standards and the educational attributes of educations (Shen, 2015). However, established serious game evaluations are mostly limited to educational and medical fields, and their applicability in the cultural heritage domain remains unverified. Therefore, the research on a CH-SGs evaluation rubric in this paper aims to fill this gap, promoting the development of both theoretical and empirical research on CH-SGs evaluation. It also aids other types of serious games in exploring suitable evaluation indicators and assessment tools. This study posits that with the guidance of evaluation rubrics, the design process of CH-SGs is akin to having a measuring ruler and a direction, leading to breakthroughs in research on design, development, and application evaluations of CH-SGs (Wang et al., 2019a). An evaluation rubric is a scoring tool that includes standards for assessing a task, encompassing various indicators each with its specific weight and assessment criteria. It is a scoring tool that can present indicators in the form of charts or tables. The Chinese scholar Li and Zhu (2017) considers rubrics as structured quantitative evaluation tools, useful for assessing student digital works with strong operability and accuracy, allowing evaluations by teachers, students, and peers (Li and Zhu, 2017). The evaluation rubric in this paper includes various levels of indicators based on theoretical foundations, representing a comprehensive set of evaluation indicators. It revolves around the needs of CH-SGs users, serving as a reference for designers. Establishing a CH-SGs evaluation rubric requires more than a single theoretical construction; it necessitates examination from the user’s perspective (Xu L. Q., 2019). However, it is observed that the existing evaluation rubrics are predominantly used by teachers, designers, and parents, with few specifically formulated for users. The primary evaluators of these games are educators and developers, not the learners or users. This study argues that the users are the true evaluators of serious games, which requires developers to consider game content that matches user needs right from the design stage (Shen and Zhang, 2014). However, accurately grasping user needs is challenging, and past qualitative evaluations based on user experience have overly relied on the evaluators’ quality, often being subjective and lacking scientific rigor (Jin and Yang, 2017). Therefore, this study attempts a multidimensional exploration of user need indicators and a multi-method validation of the evaluation rubric.

**Figure 2:**
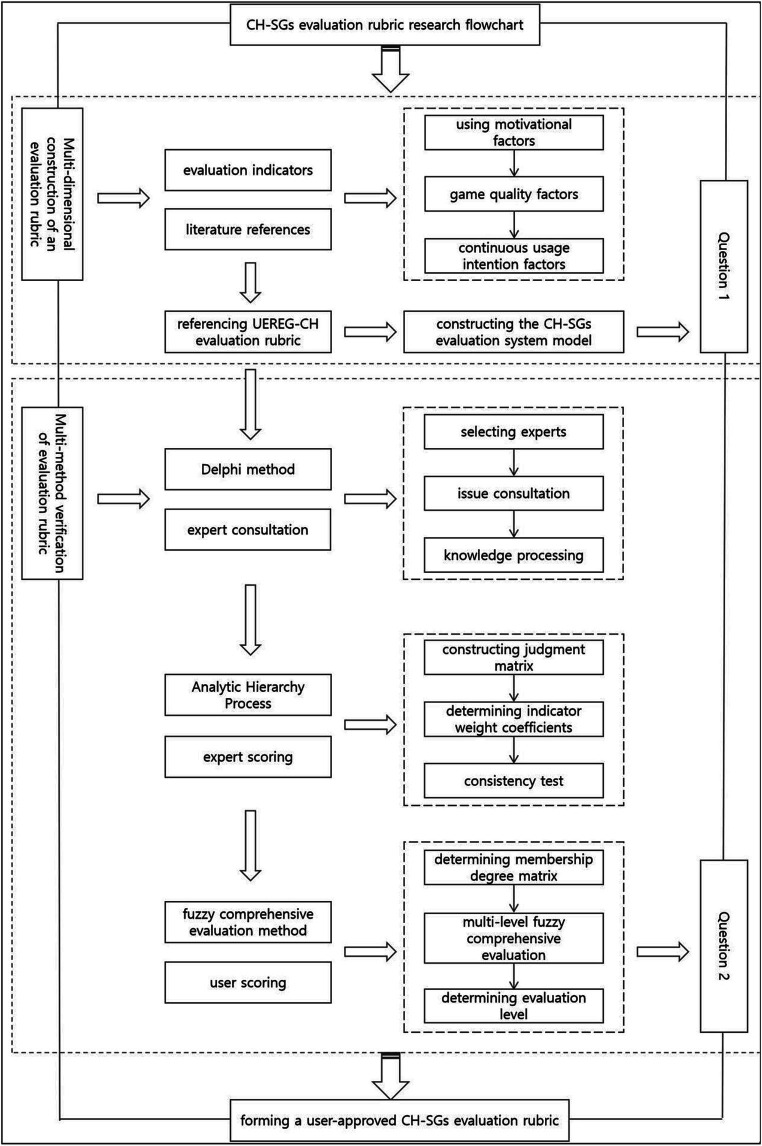
Research flowchart.

In summary, this research focuses on two main questions: how to construct a multi-dimensional CH-SG evaluation rubric that meets user needs, and whether the constructed CH-SG evaluation rubric is accepted by users. This article summarizes 14 user demand factors through literature induction, from the perspectives of usage motivation, game quality, and continuous usage intention; refers to the User Experience Rubric for Educational Games-CH (UEREG-CH) evaluation scale to initially form a CH-SG evaluation system model with 6 primary indicators, 14 secondary indicators, and 32 tertiary indicators; revises and perfects this model in terms of dimensions and semantics through the Delphi expert consultation method, expanding the original 32 tertiary indicators to 38; then conducts Analytic Hierarchy Process (AHP) hierarchical analysis with expert scoring to assign weights to each indicator of the model; subsequently, through user questionnaire surveys, scores are assigned to this model, combined with the weights given by experts to perform fuzzy comprehensive calculations, obtaining the final user evaluation value to determine whether users accept the CH-SG evaluation scale (Figure 2).

This study constructs the evaluation rubric for Cultural Heritage Serious Games (CH-SGs) from three dimensions: usage motivation, game quality, and continuous usage intention. Initially, one issue with CH-SGs is that they fail to prioritize users’ motivational needs (Tang, 2022). Instead, from design to content, these games often align with the aesthetics of designers and professionals, as evidenced by games like “Tenon and Mortise,” awarded as an excellent app by the Apple App Store in 2014, “Folding Fan” winning the “2018 Zcool Award - Interface Design Category” special award, and “Nishan Shaman” receiving the “Lina and Haifeng, 2020 China Best Sound Effect Award” among others. While these games are recognized in the industry, they struggle to captivate users, often described as “one-time games” or “beautiful but useless,” indicating that designers are not fully aware of the real motivations of users. In terms of usage motivation literature, Chinese research on game users, especially focusing on the psychological motivations of adolescents using online games, is limited (Chen, 2023). The scholar Zhong Zhijin found that players engaged in online games are influenced by different motivations like personal achievement, enjoying a social life, and immersing in virtual worlds to escape reality, all significantly boosting game duration and stickiness (Zhong, 2011). Research by Ye Na, involving an empirical survey of 546 college students playing online games, showed that the relationship between achievement satisfaction and online game addiction was the strongest (Ye et al., 2009). This study references Zhang Wenmin’s research on Multiplayer online battle arena (MOBA) mobile game users’ continuous usage intentions, which posits that social, competitive, immersive, and cognitive motivations significantly impact users’ continued usage (Zhang, 2019). Furthermore, the scholar Holbrook noted that the product provided by online games is virtual, offering psychological and emotional satisfaction through emotional interaction with others, leading to leisure enjoyment (Holbrook and Hirschman, 1982). In her empirical study on the relationship between customer perceived value, satisfaction, and loyalty in online games, Hu (2013) also found that leisure enjoyment positively impacts customer retention. Therefore, based on the above discussions, this study incorporates cognitive satisfaction, immersive satisfaction, achievement satisfaction, social satisfaction, and leisure satisfaction into the user motivation indicators for measurement, defined as follows:

1. Cognitive satisfaction: Kahn believes this involves helping users develop specific intellectual abilities through games.
2. Immersive satisfaction: Yee defines this as the behavior or psychological state where users immerse themselves in the virtual world of the game, escaping reality.
3. Achievement satisfaction: This refers to the user’s strong desire to achieve victory in the game.
4. Social satisfaction: Yee sees this as the user’s expectation to achieve social activities on the gaming platform.
5. Leisure satisfaction: Holbrook suggests that through emotional interaction in online games, customers experience a sense of well-being in their consciousness, meeting their psychological and emotional needs and expectations.

Secondly, as products of the internet era, CH-SGs require support from various sectors of the industry chain, from pre-launch advertising by game operators, provision of downloads by distributors, and stable service from service providers to continuous updates and optimization by developers (Jiang, 2011). Users may choose to continue or abandon a game due to various factors such as server quality and system stability. A prominent issue in the current design of CH-SGs is the simplicity of their mechanism design, lacking in gameplay and intrigue, and their weak playability. Additionally, the integration of educational elements with gameplay is insufficient (Lei and Feng, 2020). For instance, games like “Tenon and Mortise” and “Folding Fan” by Tag Design team are termed as “serious games,” but they focus more on conveying the artistry of cultural heritage, with more emphasis on aesthetics than on interactive gaming, failing to convey information through game mechanics or narratives, which points to a deficiency in the game quality of CH-SGs (Zhang S. Y., 2023). Among the literature related to game quality, many scholars view games as a new type of information system and have incorporated the revised model of information system success into studies on game user engagement, confirming that system quality, information quality, and service quality influence user participation (Wang, 2010). This study refers to the scholar Zhang (2016), who analyzed mobile game user churn and attributed game factors to system quality, service quality, game design, and brand image, demonstrating significant impacts on the perceived usefulness and ease of use by game users (Fang, 2023). Furthermore, CH-SGs differ from traditional online games in that they are primarily designed to address real-world social and industrial problems. They are not solely for entertainment but are meant to achieve at least one additional goal, namely, the learning objective, without compromising the user’s gaming experience (Peng and Liu, 2020). Many studies have explored different quality standards for serious games, including effectiveness and attractiveness, but have not fully considered the dual mission of serious games, i.e., achieving both functional and gaming aspects. High-quality serious games must balance functionality and gaming; they must systematically support users in achieving learning goals (functional part) and must engage and maintain entertainment objectives (gaming part). These two aspects should be perfectly matched and integrated, rather than being addressed in isolation (Caserman et al., 2020). Therefore, this paper also considers learning and entertainment objectives as factors in game quality, defined as follows:

1. System quality: This refers to the stability and usability of the game system, essential for ensuring a normal gaming experience for users.
2. Service quality: This assesses the quality of services provided by companies to users, ensuring a comfortable and enjoyable gaming experience.
3. Game design: This involves the rational organization and arrangement of various elements of a game product, creation through game engines, and the final formation of the game product.
4. Brand image: The perceived influence of the game brand by users, including the brand image of the game itself and the company’s reputation.
5. Learning objectives: The learning objectives of CH-SGs should be closely related to their application fields. The learning or training effect should remain the focus throughout the game process, always supporting users in achieving this purpose, with game elements not interfering with the learning or training process.
6. Entertainment objectives: High-quality CH-SGs should be engaging and enjoyable, focusing on user involvement, positive gameplay experiences, fluidity, and feeling of control, as well as supporting social interaction to ensure users find enjoyment in the game.

Additionally, this study emphasizes the promotion of continued usage of CH-SGs. Parthasarathy once noted in the context of information systems that retaining an existing customer costs five times less than acquiring a new one (Yu and Cheng, 2012). Long-term user feedback helps understand the ever-changing needs of users, improving and innovating game products. Continued usage intention is defined as the subjective desire of users to continue using an information system after initial use (Niu, 2020). In the field of continued usage intention, the technology acceptance model (TAM) has been widely applied and has become one of the most mature theories in this area (Sun, 2023). As online games, fundamentally, belong to the category of information systems (Li, 2011), many scholars have used the TAM model in their studies of online game users’ continuous usage (Min, 2018). The TAM model predicts the degree to which a person accepts, uses, or rejects information technology, effectively explaining the behavior of users in adopting new technologies and its influencing factors (Sun and Guo, 2023), and is the most concise and universal model for studying the initial intention and continuous intention to adopt technology (Yao, 2023). Davis (1989) first proposed the TAM model while studying users’ acceptance of computers. He suggested that perceived usefulness is an extent indicator, reflecting the individual’s perception of how an information system enhances their work efficiency. Higher perceived usefulness increases the likelihood of taking actions; perceived ease of use reflects the individual’s perceived difficulty in using the system, with higher ease of use leading to more likely user behavior (Jia, 2019). Later in 2000, Davis and Venkatesh revised the TAM to include subjective norms based on numerous studies (Dong, 2017). Scholars like Sun Shaojun integrated the TAM and Stimulus-Organism-Response (SOR) models to establish a continuous usage behavior model for game live streaming (Sun and Zhang, 2017); Wu Xuanying integrated the TAM model and flow theory to construct a stickiness influence model for mobile game users (Wu, 2019); Zhang Di combined the SOR, TAM, information success model, and immersion theory to build a model for mobile game user churn factors (Zhang, 2016). These studies confirmed the positive impact of perceived usefulness, ease of use, and subjective norms on continuous usage. Therefore, this study incorporates these three variables into the continuous usage indicators, defined as follows:

1. Perceived usefulness: The degree to which a user perceives that using a particular information system will help them perform tasks better or gain other benefits. When users perceive that using such games meets their expected benefits, they will feel satisfied and continue using the games.
2. Perceived ease of use: The degree to which a user perceives the ease or difficulty of using a particular information system. If users encounter difficulties when experiencing a game, they may feel dissatisfied and abandon it, whereas if they find the game easy to operate, they will feel satisfied and continue using it.
3. Subjective norms: The degree to which a user’s behavior is influenced by group dynamics, such as conformity, suggestion, and obedience. Therefore, an individual’s behavior is often influenced by the group. If users perceive that no one in their social circle is using the game, they may feel that the game is futile and abandon it due to a lack of positive gaming experience.

## 2 Methodology

### 2.1 Constructing the CH-SG evaluation system model

This study posits that only by establishing an evaluation rubric fitting the CH-SG context can effective assessments be conducted in all aspects, referencing the UEREG-CH evaluation scale with high similarity prior to construction (Table 1). This scale was developed by the team at the Educational Game Center of Nanjing Normal University. It is guided theoretically by Hartmann’s improved model of user experience influencing factors, reviews the current state of research on factors influencing user experience, and analyzes the characteristics and elements of serious game user experiences. The rubric identifies key terms in user experience evaluation through user testing, and then refines the primary and secondary indicators of the index system through two rounds of expert consultation, thus ultimately determining this evaluation rubric (Shen, 2015).

**Table 1:** The UEREG-CH evaluation rubric.

**Primary indicator**	**Secondary indicator**	**Description**	**Excellent**	**Good**	**Average**	**Poor**
Perceived aesthetics 0.1039	Clear interface design 0.2728	I can clearly see the words and images on the interface				
	Ordered menu arrangement 0.1987	I can quickly find the command buttons I need				
	Innovative and interesting interface elements 0.1572	I find the game interface elements innovative and interesting				
	Refined interface production 0.1295	I find the interface clear and realistic				
	Pleasant interface feel 0.2425	I feel very pleasant when I see the interface				
Usability 0.2547	Easy to remember 0.0843	I can remember the meanings of symbols in the game				
	Easy to operate 0.1766	The game operations are handy, and the button placement is reasonable				
	Easy to learn 0.1644	I can quickly learn how to use this game				
	Stable game operation without faults 0.3288	There are no bugs during game use				
	Clear and timely system feedback 0.2462	I can know the result of each of my actions through system feedback				
Educational value 0.2449	Clear and timely knowledge feedback 0.1392	I can timely know the correctness of the knowledge I learn from the game				
	Level design compliant with learning rules 0.2588	The game levels are designed in line with cognitive rules and general life logic				
	Reliable content with flexible and varied forms 0.1909	The knowledge content in the game is accurate and reliable, presented in various forms				
	Clear learning objectives 0.2060	I can clearly know my learning objectives				
	Balance between skills and challenges 0.2051	I can complete this game through my efforts				
Gamification 0.1764	Moderate challenge 0.2423	The challenges in the game stimulate my eagerness to learn				
	Reasonable incentives 0.1984	I am satisfied with the rewards I get in this game				
	Optional content 0.1083	I can choose the learning content				
	Attractive plot 0.2092	I find the game plot very attractive				
	Clear game rules 0.2419	I can clearly understand the rules of this game				
Needs 0.2201	Learning content meets or exceeds learner needs 0.5123	I can easily master the knowledge points in this game				
	Game functionality meets or exceeds learner needs 0.4877	The learning functions in the game meet my needs for learning this knowledge				

This paper, based on literature research, identifies 14 influencing factors: ‘Intellectual satisfaction, Immersion satisfaction, Achievement satisfaction, Social satisfaction, Pleasure satisfaction, System quality, Service quality, Game design, Brand image, Learning objectives, Entertainment objectives, Perceived usefulness, Perceived ease of use, and Subjective norms’. These factors are used as secondary indicators in the CH-SG evaluation system model. Referring to the dimensions in the UEREG-CH evaluation rubrics, these 14 secondary indicators are categorized into 6 primary indicators and further expanded into 32 tertiary indicators, thereby preliminarily forming the CH-SG evaluation system model (Liu et al., 2021) (Table 2).

**Table 2:** Initial establishment of the CH-SG evaluation system model.

CH-SGs evaluation system model			
CH-SG Evaluation System Model	B1 Artistic dimension	C1 Game design	D1 Game interface operation setting is reasonable, with the characteristics of the cultural heritage theme
			D2 Creative game content design, engaging character roles, and cultural skill settings
			D3 High clarity and realistic, engaging game graphics
		C2 Immersion satisfaction	D4 Immersed in the game’s artistic roles, creating one’s own story background
			D5 Immersing oneself in the game’s story to concentrate attention, thereby avoiding thinking about problems in real life
	B2 Usability	C3 System quality	D6 Stable game system, bug-free, regular updates, and optimization
			D7 Effective game guidance, helping users access necessary game information
		C4 Service quality	D8 Regular promotional activities by the game manufacturer and fulfillment of promises to users
			D9 Competent customer service with good knowledge and timely problem resolution
	B3 Educational value	C5 Intellectual satisfaction	D10 Gaining knowledge of traditional culture through learning tasks in the game
			D11 Enhancing intellectual abilities through completing tasks and teamwork
		C6 Perceived usefulness	D12 The game helps in life and study, relieving mental stress
			D13 Enriching leisure life and stimulating curiosity to learn traditional culture
		C7 Learning objectives	D14 Continuous and systematic design of learning content in the game, clear learning objectives, and undisturbed learning process
			D15 Identifiable multi-modal synchronous feedback during the learning process, active and diverse positive rewards
			D16 Accurate and reliable cultural heritage knowledge in the game, diverse presentation, and cognitively appropriate
	B4 Gamification	C8 Perceived ease of use	D17 Ease of downloading, installing, and paying for the game
			D18 Ease of learning and mastering the game operations
		C9 Entertainment objectives	D19 Engaging multi-modal experiences in the game, empathetic emotional responses between the user and characters through graphics, sound, and tactile experiences
			D20 In the game, the difficulty matches the player’s level, and the user has control and influence. The user can interact with others in modes of competition, collaboration, etc.
			D21 Fulfilling users’ creative and self-expression needs through the game
			D22 Relaxing and pleasurable mental experiences from playing the game
		C10 Pleasure satisfaction	D23 New experiences in the game relieve stress and enrich scattered and short periods of time in life while increasing conversation topics
			D24 Participating in offline experiences and purchase of traditional culture-related products to beautify life
	B5 Needs	C11 Social satisfaction	D25 Collaborating and communicating with other users in the game, making many friends
			D26 Maintaining long-term contact with friends through the game, feeling care and support among friends
		C12 Achievement satisfaction	D27 Desire to challenge and compete with others in the game, valuing victory in the game
			D28 Rapid progression of the game character by mastering the rules, achieving wealth and status in the game
	B6 Identity	C13 Brand image	D29 High recognition of the game or its cultural IP theme that I like
			D30 High recognition of the game production company or use by experts, celebrities, etc., whom I like
		C14 Subjective norms	D31 Many people around me play this game and recommend it to me
			D32 Recommendations for the game by experts, media, or important people in my life

### 2.2 The Delphi method

The Delphi method is a group decision-making behavior that involves a series of concentrated expert surveys. Based on the collective professional knowledge and experience of experts, supplemented by controlled feedback of opinions, the process aims to achieve the greatest consensus among a group of experts (Dai, 2023). The advantage of the Delphi method is that it avoids direct confrontation between experts and structures the discussion process of the expert group, making them more effective when discussing complex issues (Liu et al., 2021). It is not only used in the field of prediction but also widely used in the establishment of various evaluation systems and the determination of specific indicators (Liu et al., 2011). The 10 experts who filled out the questionnaire on game studies were strictly selected and all had at least 2–3 years of research experience in serious games, educational games, and online games, with at least 2 years of game testing experience and at least one experience in educational game evaluation (Liu et al., 2023).

Ten experts were consulted over two rounds regarding the initially established CH-SGs evaluation system model, using both electronic mail and paper questionnaires to collect expert opinions and to add, delete, or modify indicators. The specific process included: clarifying evaluation objectives, selecting experts, issuing questions, experts’ assessment of questions, and revising based on expert feedback (Liu et al., 2011). The first round of questionnaires involved experts’ familiarity with each level of indicators, suggestions for additional or deletable indicators, and other questions or recommendations. Based on expert suggestions, similar indicators in the CH-SG evaluation system model were merged, unreasonable ones were deleted or broken down, and missing indicators were added (Dong, 2013). In the first round of questionnaire consultation, the primary indicator “Identity dimension” was not described accurately and was loosely connected logically with the secondary indicators “Brand image” and “Subjective norms,” hence it was changed to “Social dimension.” The secondary indicator “Game design” was too broad and encompassed too much, so it was changed to “Interface design” based on the tertiary indicators. Complex descriptions in tertiary indicators were further refined, such as “D1 Game interface operation setting is reasonable, with the characteristics of the cultural heritage theme,” which was split into “D1 Interface design features cultural heritage theme” and “D2 Interface menu layout is reasonable and orderly” for easier understanding and scoring by experts or users. “D4 Immersed in the game’s artistic roles, creating one’s own story background” was simplified to “D5 Immersion in creating one’s story with artistic roles,” etc. Other less comprehensive descriptions were expanded, thus increasing the original 32 tertiary indicators to 38. Overall, the experts acknowledged the feasibility of the indicators, with minimal modifications made (Table 3).

**Table 3:** Revised CH-SG evaluation system model.

CH-SG Evaluation System Model	B1 Artistic dimension	C1Interface design	D1 Interface design features cultural heritage theme
			D2 Interface menu layout is reasonable and orderly
			D3 Creative character roles and cultural skill settings
			D4 High clarity, exquisite realism, and interesting visual scenes
		C2 Immersion satisfaction	D5 Immersing in creating one’s story with artistic roles
			D6 Helping to forget the problems and stresses of real life
	B2 Usability	C3 System quality	D7 Stable game system, bug-free, regular updates, and optimization
			D8 Effective game guidance, helping users access necessary game information
			D9 Synchronous feedback mode is diverse, timely, and recognizable
		C4 Service quality	D10 Regular promotional activities by the game manufacturer and fulfillment of promises to users
			D11 Customer service staff being competent and promptly resolving user issues
	B3 Educational value	C5 Learning objectives	D12 The learning content is continuous and systematic
			D13 The learning objectives are clear, rigorous, and not distracted by games
			D14 Knowledge feedback is positive and multi-formally rewarding
			D15 Cultural heritage knowledge is accurate, reliable, and varied in form
			D16 Level design conforms to learning laws and user cognitive levels
		C6 Intellectual satisfaction	D17 Enhancing the ability to complete tasks and cooperate with others intellectually
			D18 Stimulating curiosity to learn traditional culture
		C7 Perceived usefulness	D19 Helping in life and study, relieving mental stress
			D20 Enhancing the level of traditional cultural knowledge and skills
	B4 Gamification	C8 Perceived ease of use	D21 Ease of downloading, installing, and paying for the game
			D22 Ease of learning and mastering the game operations
		C9 Entertainment objectives	D23 Establishing multi-modal experiences such as graphics, sound effects, and tactile
			D24 Able to highly concentrate attention and empathize with characters
			D25 Can control to affect game difficulty or plot direction
			D26 Realizing self-creation, self-expression, exploration, and discovery
			D27 Obtaining pleasure, mental enjoyment, relaxation, and immersion
		C10 Pleasure satisfaction	D28 Enrich scattered and short periods of time in life while increasing conversation topics
			D29 Participating in offline experiences and purchase of traditional culture-related products
	B5 Needs	C11 Social satisfaction	D30 Can choose games with different modes like interaction, competition, collaboration with others
			D31 Making many friends through cooperation and communication with other users
			D32 Maintaining long-term contact with friends through the game, feeling care and support among friends
		C12 Achievement satisfaction	D33 Desire to challenge and compete with others in the game, valuing victory in the game
			D34 Rapid progression of the game character by mastering the rules, achieving wealth and status in the game
	B6 Social dimension	C13 Brand image	D35 High recognition of the game or its cultural IP theme that I like
			D36 High recognition of the game production company or use by experts, celebrities, etc., whom I like
		C14 Subjective norms	D37 Many people around me play this game and recommend it to me
			D38 Recommendations for the game by experts, media, or important people in my life

The revised CH-SG evaluation system model consists of 6 primary indicators, 14 secondary indicators, and 38 tertiary indicators. In the second round of questionnaire surveys, the opinions of the experts converged, with the agreement rate for each level of indicators reaching 100%. Therefore, no further modifications were needed, and the CH-SG evaluation system model was finally determined to be used as the AHP for the next phase of research.

### 2.3 AHP hierarchical analysis

The AHP is a decision-making method for complex and vague issues, particularly suitable for issues difficult to be fully quantified. It is a flexible, convenient, and practical multi-criteria decision-making method (Zhang et al., 2014). The principle of AHP is to organize and hierarchize problems, constructing a structured model with various levels. Complex issues are decomposed into elements, which are then grouped according to their characteristics and certain rules into several levels. Elements within each level have approximately the same status. An orderly, step-by-step hierarchy is established based on the affiliation relationships between levels, where elements of a higher level dominate related elements of the next lower level (Dong, 2013). In this step-by-step hierarchical model, decision-makers score the importance of each level quantitatively based on their judgment of certain objective facts. This is done by determining the relative importance of elements within each level through pairwise comparison and quantitatively expressing this importance, thereby establishing judgment matrices. Mathematical models are then used to calculate the relative importance weights of each indicator within the judgment matrices of each level. Finally, the relative importance weights of all indicators concerning the evaluation objective are calculated through corresponding operations (Xu, 2009). In the CH-SG hierarchical model of this paper, the relationship between primary, secondary, and tertiary indicators is one of containment and being contained, also known as the upper and lower level relationship in the decision-making field. In the hierarchical model, “CH-SG Evaluation System Model” A is the goal layer, B1-B6 are primary indicators, C1-C14 secondary indicators are criterion layers, and D1-D38 tertiary indicators are the scheme layer. Experts compare and score the indicators in the evaluation system (Table 4), generate judgment matrices, and calculate the weight values of each indicator.

**Table 4:** Expert questionnaire scoring 1–9 scale method.

**aij**	**Definition**	**aij**	**Definition**
1	Ai and Aj are equally important	2	Between equally important and slightly more important
3	Ai is slightly more important than Aj	4	Between slightly and significantly more important
5	Ai is significantly more important than Aj	6	Between significantly and very significantly more important
7	Ai is very significantly more important than Aj	8	Between very significantly and absolutely more important
9	Ai is absolutely more important than Aj	Inverse	aij is the result of comparing the importance of indicators i and j，aij = 1/aji

Importance comparison evaluation table.

Steps for calculating weights using the AH involve the hypothetical judgment matrix A:


A=a11a12⋯a1na21a22⋯a2n⋯⋯⋯⋯an1an2⋯ann

The calculation steps for the root method are as follows:

(1) Calculate the product of the elements in each row of the judgment matrix A:


$$m_{t}=\underset{j=1}{\prod }a_{ij},i=1,2\cdots n$$

(2) Calculate the nth root of mi;


$$w_{i}^{*}=\sqrt[{n}]{m_{i}}$$

(3) Normalize the vector $W^{*}={\left(w_{1}^{*},w_{2}^{*},\cdots ,w_{n}^{*}\right)}^{T}$


$$w_{i}=\frac{w_{i}^{*}}{\overset{n}{\underset{i=1}{\sum }}w_{i}^{*}}$$

The weights obtained are the desired weight vector, w1, w2, ..., wn, which corresponds to the weight value of each element.

Matrix consistency test method:

(4) Calculate the consistency index


$$CI=\frac{\lambda_{\text{max}}-n}{n-1}$$

The calculation formula for the largest eigenvalue is:$\lambda_{\text{max}}$


$$\lambda_{\text{max}}=\frac{1}{n}\overset{n}{\underset{i=1}{\sum }}\frac{{\left(\mathrm{Aw}\right)}_{i}}{w_{i}}$$

(5) Look up the corresponding Average Random Consistency Index RI.

**Table:** 

*n*	1	2	3	4	5	6
RI	0	0	0.58	0.90	1.12	1.24
*n*	7	8	9	10	11	12
RI	1.32	1.41	1.45	1.49	1.52	1.54

(6) Average Consistency Ratio (CR).


$$CR=\frac{CI}{RI}$$

When CR is less than 0.1, the consistency of the judgment matrix A is considered acceptable; otherwise, some adjustments to the judgment matrix are required (Zhu et al., 2020) (Refer to the appendix for the detailed calculation process).

Based on the scoring from 10 experts, a judgment matrix is constructed, and the results of the weight calculation using the AHP are as follows:

Table 5 shows that the CR value is <0.1, passing the consistency test. The smaller the CR value, the better the consistency of the judgment matrix, indicating the rationality of this weight determination method. There is no need to modify the judgment matrix. The derived weight set can reflect the importance of each indicator, and the weight distribution is reasonable (Li and Guo, 2004).

**Table 5:** Results of the consistency test.

**Evaluation index**	**Λmax**	**CI**	**RI**	**CR**
B1	6.4380	0.0876	1.24	0.0707
B2				
B3				
B4				
B5				
B6				
C1	2.0000	0.0000	0	0.0000
C2				
C3	2.0000	0.0000	0	0.0000
C4				
C5	3.0536	0.0268	0.58	0.0462
C6				
C7				
C8	3.0000	0.0000	0.58	0.0000
C9				
C10				
C11	2.0000	0.0000	0	0.0000
C12				
C13	2.0000	0.0000	0	0.0000
C14				
D1	4.1171	0.0390	0.9	0.0434
D2				
D3				
D4				
D5	2.0000	0.0000	0	0.0000
D6				
D7	3.0536	0.0268	0.58	0.0462
D8				
D9				
D10	2.0000	0.0000	0	0.0000
D11				
D12	5.2758	0.0689	1.12	0.0616
D13				
D14				
D15				
D16				
D17	2.0000	0.0000	0	0.0000
D18				
D19	2.0000	0.0000	0	0.0000
D20				
D21	2.0000	0.0000	0	0.0000
D22				
D23	5.1398	0.0349	1.12	0.0312
D24				
D25				
D26				
D27				
D28	2.0000	0.0000	0	0.0000
D29				
D30	3.0536	0.0268	0.58	0.0462
D31				
D32				
D33	2.0000	0.0000	0	0.0000
D34				
D35	2.0000	0.0000	0	0.0000
D36				
D37	2.0000	0.0000	0	0.0000
D38				

Through the above research, the weight coefficients of each level of indicators for the CH-SGs evaluation were determined (Table 6), forming a complete evaluation system. Subsequently, the fuzzy comprehensive evaluation method will be used by users to assess the overall quality of this system (Zhang X. W., 2023).

**Table 6:** Weight values of each indicator in the CH-SG evaluation system model.

**Criterion layer**	**Relative weight**	**Sub-criterion Layer**	**Relative weight**	**Indicator layer**	**Relative weight**	**Absolute weight**
B1 Artistic dimension	0.22546	C1 Interface design	0.666667	D1 Interface design features cultural heritage theme	0.286955	0.043131
				D2 Interface menu layout is reasonable and orderly	0.2413	0.036269
				D3 Creative character roles and cultural skill settings.	0.154177	0.023174
				D4 High clarity, exquisite realism, and interesting visual scenes	0.317568	0.047733
		C2 Immersion satisfaction	0.333333	D5 Immersing in creating one’s story with artistic roles	0.75000	0.056365
				D6 Helping to forget the problems and stresses of real life	0.25000	0.018788
B2 Usability	0.178948	C3 System quality	0.666667	D7 Stable game system, bug-free, regular updates, and optimization	0.493386	0.05886
				D8 Effective game guidance, helping users access necessary game information	0.1958	0.023359
				D9 Synchronous feedback mode is diverse, timely, and recognizable	0.310814	0.03708
		C4 Service quality	0.333333	D10 Regular promotional activities by the game manufacturer and fulfillment of promises to users	0.666667	0.039766
				D11 Customer service staff being competent and promptly resolving user issues	0.333333	0.019883
B3 Educational value	0.142031	C5 Learning objectives	0.493386	D12 The learning content is continuous and systematic	0.330292	0.023146
				D13 The learning objectives are clear, rigorous, and not distracted by games	0.165146	0.011573
				D14 Knowledge feedback is positive and multi-formally rewarding	0.125157	0.008771
				D15 Cultural heritage knowledge is accurate, reliable, and varied in form	0.189703	0.013294
				D16 Level design conforms to learning laws and user cognitive levels	0.189703	0.013294
		C6 Intellectual satisfaction	0.1958	D17 Enhancing the ability to complete tasks and cooperate with others intellectually	0.666667	0.01854
				D18 Stimulating curiosity to learn traditional culture	0.333333	0.00927
		C7 Perceived usefulness	0.310814	D19 Helping in life and study, relieving mental stress	0.666667	0.02943
				D20 Enhancing the level of traditional cultural knowledge and skills	0.333333	0.014715
B4 Gamification	0.22993	C8 Perceived ease of use	0.5	D21 Ease of downloading, installing, and paying for the game	0.666667	0.076643
				D22 Ease of learning and mastering the game operations	0.333333	0.038322
		C9 Entertainment objectives	0.25	D23 Establishing multi-modal experiences such as graphics, sound effects, and tactile	0.342639	0.019696
				D24 Able to highly concentrate attention and empathize with characters	0.088621	0.005094
				D25 Can control to affect game difficulty or plot direction	0.157975	0.009081
				D26 Realizing self-creation, self-expression, exploration, and discovery	0.17132	0.009848
				D27 Obtaining pleasure, mental enjoyment, relaxation, and immersion	0.239446	0.013764
		C10 Pleasure satisfaction	0.25	D28 Enrich scattered and short periods of time in life while increasing conversation topics	0.666667	0.038322
				D29 Participating in offline experiences and purchase of traditional culture-related products	0.333333	0.019161
B5 Needs	0.105364	C11 Social satisfaction	0.5	D30 Can choose games with different modes like interaction, competition, collaboration with others	0.493386	0.025993
				D31 Making many friends through cooperation and communication with other users	0.310814	0.016374
				D32 Long-term contact with other users and feeling care among friends	0.1958	0.010315
		C12 Achievement satisfaction	0.5	D33 The desire to win in challenges and competitions	0.666667	0.035121
				D34 Rapid progression of the game character by mastering the rules, achieving wealth and status in the game	0.333333	0.017561
B6 Social dimension	0.118267	C13 Brand image	0.666667	D35 High recognition or liked cultural IP themes	0.666667	0.052563
				D36 Production companies with high recognition or liked experts and stars	0.333333	0.026282
		C14 Subjective norms	0.333333	D37 Many people around me play this game and recommend it to me	0.7500	0.029567
				D38 Recommendations for the game by experts, media, or important people in my life	0.2500	0.009856

### 2.4 Fuzzy comprehensive calculation

Fuzzy comprehensive calculation is based on fuzzy mathematics and applies the principle of fuzzy relation synthesis to quantify factors with unclear boundaries and difficult quantification, conducting a comprehensive evaluation of the belonging level of an entity under evaluation from multiple factors (Ge and Sun, 2014). The basic procedure is to first determine the factor domain of the evaluation object, i.e., the number of indicators, and second to determine the evaluation level domain, typically taking integers, and for the convenience of expert judgment, the levels are divided as finely as possible (Li and Deng, 2019). This study adopts an evaluation set of levels: poor, below average, average, good, and excellent. Finally, the weights of each indicator obtained from the AHP are combined to calculate the final fuzzy evaluation result (Wang, 2017). The fuzzy comprehensive calculation is as follows:

1 First, determine the factor domain of the evaluation object (i.e., the fuzzy factor set): let there be p evaluation indicators, U = {u1, u2, ..., up}.

2 Determine the weight vector of evaluation factors and the evaluation level domain (i.e., the evaluation set): in fuzzy comprehensive evaluation, determine the weight vector of evaluation factors: W = {a1, a2, ..., ap}. The relative importance order of evaluation indicators is usually determined using the AHP or other methods to establish the weight coefficients, and normalize them before synthesis. Let the evaluation level domain (i.e., the evaluation set) be V = {v1, v2, ..., vm}, with each level corresponding to a fuzzy subset, i.e., the level set.

3 Establish a fuzzy relation matrix (i.e., the membership degree matrix): after constructing the level fuzzy subsets, quantify the evaluated entity from each factor ui (i = 1, 2, ..., p), i.e., determine the membership degree of the evaluated entity to the level fuzzy subsets (R|ui). Through survey questionnaires, obtain the scoring of each person for each level of the indicator, with the proportion of the number of people scoring each evaluation level to the total number of experts as the membership degree, thereby establishing the single factor fuzzy comprehensive evaluation matrix.

The membership degree matrix for a single element (R|ui) = (ri1, ri2, ..., rim), where for each level of a single element, the membership degree is: rij=cijc .

Cij represents the number of people who chose level vj for indicator i, and c represents the total number of experts participating in the evaluation.

Calculate the membership degree for p evaluation indicators to obtain the overall fuzzy relation matrix, as follows:


$$R=\left[\begin{array}{l} R|u1\\ R|u2\\ \ldots \ldots \\ R|u_{p} \end{array}\right]=\left[\begin{array}{cccc} r_{11} & r_{12} & \ldots & r_{1m}\\ r_{21} & r_{22} & \ldots & r_{2m}\\ \ldots & \ldots & \ldots & \ldots \\ r_{p1} & r_{p2} & \ldots & r_{\mathrm{pm}} \end{array}\right]$$

In this element, the element rij in the ith row and jth column represents the membership degree of a certain evaluated entity ui in terms of the factor to the level vj fuzzy subset.

4 Synthesize the fuzzy comprehensive evaluation result vector: combine W with the overall fuzzy relation matrix R of each evaluated entity to obtain the fuzzy comprehensive evaluation vector B for each evaluated entity, i.e.:

B=W*R = (a1,a2,…,ap)* $\left[\begin{array}{cccc} r_{11} & r_{12} & \ldots & r_{1m}\\ r_{21} & r_{22} & \ldots & r_{2m}\\ \ldots & \ldots & \ldots & \ldots \\ r_{p1} & r_{p2} & \ldots & r_{\mathrm{pm}} \end{array}\right]$ =(b1,b2,…,bm).

bi represents the degree of membership of the evaluated entity to the level vj fuzzy subset from an overall perspective.

5 Analyze the fuzzy comprehensive evaluation result vector: the most commonly used method in practice is the principle of the maximum degree of membership. However, in some cases, its use can be forced, leading to the loss of a lot of information, and even producing unreasonable evaluation results. This paper uses the method of weighted average to determine the membership level, allowing for the ranking of multiple evaluated entities according to their level positions (refer to the appendix for the detailed calculation process).

To verify the scientific and practical nature of the CH-SG evaluation system model discussed earlier, this paper utilizes the weights of indicators determined by the AHP and applies fuzzy comprehensive calculation to evaluate the comprehensive score of this system. For this purpose, a questionnaire targeted at users of CH-SGs is designed (refer to the appendix for details). Evaluators simply need to select the corresponding levels for each indicator on the scale based on their judgment (Zhang, 2015). A total of 400 questionnaires were distributed, with 392 valid responses collected. To ensure the reliability and validity of the questionnaire, the sample data was analyzed using the Cronbach’s alpha coefficient in SPSS, yielding an overall Cronbach’s alpha of α = 0.962, indicating good internal consistency. Exploratory factor analysis was also conducted, with a Kaiser-Meyer-Olkin (KMO) value of 0.978 and a significant level of Bartlett’s test of sphericity at 0.000, indicating significant correlations between variables and passing the test of reliability and validity. This paper analyzes the collected questionnaires, calculating the frequency of each level—poor, below average, average, good, and excellent—and the corresponding values of each indicator for fuzzy comprehensive calculation. Through these calculations, the fuzzy evaluation values of each level of indicators in the CH-SGs evaluation system are derived, providing reference for further analysis of the evaluation rubric.

The fuzzy comprehensive calculation resulted in the evaluation values for each level of indicators in the CH-SG evaluation rubric (Table 7). The overall primary level fuzzy comprehensive evaluation value was 3.4228, indicating an overall level between average and good. This suggests that the evaluation rubric is recognized by users, and the descriptions of evaluations in these games closely align with user experiences (Liang, 2019). According to the data, the primary indicators are ranked by their importance as follows: B4 Gamification at 3.5371 > B5 Demand at 3.4546 > B6 Social dimension at 3.4473 > B3 Educational value at 3.4307 > B1 Artistic dimension at 3.4223 > B2 Usability at 3.2355. The B2 Usability at 3.2355 is notably lower than the overall evaluation value of 3.4228, indicating that this indicator is not ideal and suggests a mismatch between expert and user standards in evaluating the game.

**Table 7:** Fuzzy evaluation values of each indicator in the CH-SG Evaluation Rubric.

**Evaluation target**	**Criterion layer (first-level indicators)**	**Sub-Criterion layer (second-level indicators)**	**Indicator layer (Third-level indicators)**	
CH-SG Evaluation System Model 3.4228	B1 Artistic dimension 3.4223	C1 Interface design 3.2941	D1 Interface design features cultural heritage theme	3.3112
			D2 Interface menu layout is reasonable and orderly	3.1403
			D3 Creative character roles and cultural skill settings.	2.7270
			D4 High clarity, exquisite realism, and interesting visual scenes.	3.6709
		C2 Immersion satisfaction 3.6786	D5 Immersing in creating one’s story with artistic roles	3.6378
			D6 Helping to forget the problems and stresses of real life	3.8010
	B2 Usability 3.2355	C3 System quality 3.1033	D7 Stable game system, bug-free, regular updates, and optimization	3.2092
			D8 Effective game guidance, helping users access necessary game information	2.6684
			D9 Synchronous feedback mode is diverse, timely, and recognizable	3.2092
		C4 Service quality 3.5000	D10 Regular promotional activities by the game manufacturer and fulfillment of promises to users	3.4745
			D11 Customer service staff being competent and promptly resolving user issues	3.5510
	B3 Educational value 3.4307	C5 Learning objectives 3.5879	D12 The learning content is continuous and systematic	3.7908
			D13 Users’ learning objectives are clear and rigorous and will not be interfered by game factors	3.2143
			D14 Knowledge feedback is positive and provide positive feedback on whether the knowledge is correct or not, and provide various forms of positive rewards	3.3980
			D15 Cultural heritage knowledge is accurate, reliable, and varied in form	3.8316
			D16 Level design conforms to learning laws and user cognitive levels	3.4413
		C6 Intellectual satisfaction 3.6956	D17 Enhancing the ability to complete tasks and cooperate with others intellectually	3.6939
			D18 Stimulating curiosity to learn traditional culture	3.6990
		C7 Perceived usefulness 3.0145	D19 Helping in life and study, relieving mental stress	3.1250
			D20 Enhancing the level of traditional cultural knowledge and skills	2.7934
	B4 Gamification 3.5371	C8 Perceived ease of use 3.6105	D21 Ease of downloading, installing, and paying for the game	3.6301
			D22 Ease of learning and mastering the game operations	3.5714
		C9 Entertainment objectives 3.3761	D23 Establishing multi-modal experiences such as graphics, sound effects, and tactile	3.7883
			D24 Able to highly concentrate attention and empathize with characters	3.1556
			D25 Can control to affect game difficulty or plot direction	2.7372
			D26 Realizing self-creation, self-expression, exploration, and discovery	3.1888
			D27 Obtaining pleasure, mental enjoyment, relaxation, and immersion	3.4235
		C10 Pleasure satisfaction 3.5510	D28 Enrich scattered and short periods of time in life while increasing conversation topics	3.4949
			D29 Participating in offline experiences and purchase of traditional culture-related products	3.6633
	B5 Needs 3.4546	C11 Social satisfaction 3.4373	D30 Can choose games with different modes like interaction, competition, collaboration with others	3.2321
			D31 Making many friends through cooperation and communication with other users	3.4745
			D32 Long-term contact with other users and feeling care among friends the game, feeling care and support among friends	3.8954
		C12 Achievement satisfaction 3.4719	D33 The desire to win in challenges and competitions	3.5434
			D34 Rapid progression of the game character by mastering the rules, achieving wealth and status in the game	3.3291
	B6 Social dimension 3.4473	C13 Brand image 3.5485	D35 High recognition or liked cultural IP themes	3.4490
			D36 Production companies with high recognition or liked experts and stars	3.7474
		C14 Subjective norms 3.2449	D37 Many people around me play this game and recommend it to me	3.1862
			D38 Recommendations for the game by experts, media, or important people in my life	3.4209

## 3 Results

The primary indicator B4 Gamification at 3.5371 is higher than the overall comprehensive evaluation value, indicating that enhancing gameplay positively impacts the evaluation of the game and that users are most concerned with the gameplay aspect of CH-SGs. In the secondary indicators, C8 Perceived ease of use at 3.6105 and C10 Pleasure satisfaction at 3.5510 are higher than the gamification indicators, enhancing the gamification. In the tertiary indicators, D21 Easy download, installation, payment for the game at 3.6301, D22 Easy learning and operation of the game at 3.5714, D23 Establishing multi-modal experiences such as graphics, sound effects, and tactile at 3.7883, and D29 Participation in offline experiences and purchasing cultural peripheral products at 3.6633 are all higher than the gamification, having a positive impact. Designers should consider how to make the game user-friendly, aligning with the characteristics of CH-SGs as light-function games, requiring minimal time and effort from users, facilitating knowledge acquisition, character cultivation, enjoyment, and mental pleasure, and providing different experiences in sound effects, tactile modes, and offline activities, all of which will effectively increase users’ willingness to continue using the game (Gong and Liang, 2018).

The primary indicator B5 Needs at 3.4546 is higher than the overall evaluation value, positively impacting it. In its secondary indicators, C12 Achievement satisfaction at 3.4719 is slightly higher than the demand dimension. In the tertiary indicators, D31 Making many friends through cooperation and communication with other users at 3.4745, D32 Long-term contact with other users and feeling care among friends at 3.8954, and D33 The desire to win in challenges and competitions at 3.5434 are all higher than the demand dimension, positively impacting it. Designers should focus on expanding users’ social pathways and convenience, fully satisfying users’ desires for social interaction and emotional communication, and increasing the game’s competitiveness and positive feedback to stimulate users’ sense of achievement, thereby promoting their willingness to continue using the game (Chang, 2015).

The primary indicator B6 Social dimension at 3.4473 is higher than the overall evaluation value, positively impacting it. In its secondary indicators, C13 Brand image at 3.5485 is higher than the social dimension indicator. In the tertiary indicators, D36 Production companies with high recognition or liked experts and stars at 3.7474 and D35 High recognition or liked cultural IP themes at 3.4490 are both higher than the primary indicator. This indicates that production companies with high recognition are a guarantee of high-quality games for users. Choosing well-known experts and stars for advertising endorsements can effectively attract users’ attention. Alternatively, choosing popular, well-received IPs for game development can arouse users’ curiosity and encourage continued use (Wang, 2022).

The primary indicator B3 Educational value at 3.4307 is slightly higher than the overall evaluation value, also having a promotive effect. In its secondary indicators, C5 Learning objectives at 3.5879 and C6 Intellectual satisfaction at 3.6956, are higher than the primary indicator. In the tertiary indicators, D12 Continuous and systematic learning content at 3.7908, D15 Accurate and reliable cultural heritage knowledge in various forms at 3.8316, D16 Level design conforming to learning rules and user cognitive level at 3.4413, D17 Enhancing the ability to complete tasks and cooperate intellectually with others at 3.6939, and D18 Stimulating curiosity in learning traditional culture at 3.6990, all these indicators are higher than the primary indicator. The educational value is where serious games differ from other entertainment games in value, thus gaining more attention from users (Li and Sun, 2019). Designers, while developing the educational goals of the game, must first ensure that the integrated cultural heritage knowledge is accurate and diverse, maintaining users’ freshness toward knowledge. Secondly, they must consider whether the designed cultural knowledge and skills are systematic, enabling users to learn progressively rather than a one-time experience that cannot be sustained. They must also consider whether the knowledge broken down into stages of task levels conforms to the users’ cognitive level, ensuring that users can overcome the task with some effort or seeking help, without feeling a sense of failure and giving up. Finally, users should gain from the use and learning of the game, either through increased positive rewards or by setting stage-wise tasks and evaluation rewards, ensuring that users have gains at every stage after completing tasks or cooperating with other users, and developing new cognition and curiosity about cultural heritage. These aspects can effectively stimulate users to continue using the game (Huang, 2023).

The primary indicator B1 Artistic dimension at 3.4223 is close to the overall evaluation value. In its secondary indicators, C2 Immersion satisfaction at 3.6786 is higher than the artistic dimension, positively impacting it. In the tertiary indicators, D4 High clarity, exquisite realism, and interesting visual scenes at 3.6709, D5 Immersion in creating one’s own artistic characters and stories at 3.6378, and D6 Helps to forget problems and stresses of real life at 3.8010, all these indicators are higher than the primary indicator value. This suggests that users hope to immerse themselves in a virtual world through the game, temporarily escaping life’s troubles for mental rest. To achieve such immersion (Wang et al., 2019b), designers need to enhance the visual effects of game scenes and endow characters with artistry, strongly attracting users’ attention, and giving them more control to create their own characters and stories (Zhang, 2018). Stimulating these aspects can enhance users’ willingness to continue using such games.

The primary indicator B2 Usability at 3.2355 is below the overall evaluation value, indicating that this indicator is not ideal and that there is a mismatch between experts’ and users’ standards in evaluating the game. In its secondary indicators, C4 Service quality at 3.5000 is higher than both the primary indicator and the overall evaluation value, suggesting that users are more concerned about the service attitude and quality of the game production company. In its tertiary indicators, D10 Conducting promotional activities and fulfilling promises to users at 3.4745 and D11 Customer service staff being competent and promptly resolving user issues at 3.5510, these indicators indicate that if game production companies deceive users during use or fail to fulfill various promotional promises, or if customer service staff have a poor attitude, lack professional competence, or do not promptly resolve users’ issues in the game, it will lead to user dissatisfaction and the desire to quit the game.

## 4 Discussion

### 4.1 Comparative analysis of CH-SGs and UEREG-CH setting principles

This section compares and analyzes the CH-SGs evaluation model and UEREG-CH based on three principles of rubric setting: purposefulness, systematicity, and measurability. Purposefulness is the fundamental basis for establishing evaluation rubrics and directly affects the conduct of evaluation activities. Systematicity requires that the evaluation rubric should comprehensively reflect the requirements of the evaluation objective, without missing any significantly important indicators. Additionally, indicators should be mutually independent, without causal or overlapping relationships, clearly hierarchical, and collectively form an organic whole. Measurability demands that specific indicators should be defined in operationalizable language, allowing for conclusions to be drawn through actual observation or measurement. This principle concretizes and quantifies abstract evaluation objectives, and is one of the basic conditions for the practical application of evaluation rubrics and the generation of reliability (Shen, 2015).

Purposefulness: The UEREG-CH primarily reflects the sensory experiences and real needs of serious game users, including students and teachers. The purpose of the evaluation is to select educational games that meet the autonomous learning needs of learners and adapt to different learning styles. The CH-SGs in this study primarily focuses on user needs and experiences to promote continued use. It provides scientific and reasonable guidance and evaluation standards for designers and developers of these games and assists users and learners in selecting suitable CH-SGs.

Systematicity: The UEREG-CH, centered on user experience as the evaluation objective, sets up 5 primary indicators, 22 secondary indicators, and corresponding descriptions and weights, with a tightly interconnected hierarchical relationship. For example, under “Gamification,” there are secondary indicators like “Appropriate challenge” and “Reasonable incentives.” However, a shortcoming is the similarity or lack of distinction between some secondary indicators, such as “Organized interface menu” and “Clear interface design”; and “Easy to remember” and “Easy to learn.” CH-SGs in this study sets up 6 primary indicators, 14 secondary indicators, and 38 tertiary indicators, assigning weights to each. Focusing on user experience, it emphasizes promoting continued use and includes content related to user motivation and game quality. The considered range of indicators is broader, deeper, and more detailed, with each level of indicators being interrelated yet distinct and easy to differentiate.

Measurability: Both the UEREG-CH and CH-SGs achieve clarity and conciseness in the design of indicator descriptions. In designing the scale method, descriptive terms like “excellent,” “good,” “average,” “poor,” and “below average” are used. Evaluators score according to the degree to which the last level of indicators meets the description.

### 4.2 Comparative analysis of CH-SGs and UEREG-CH indicator dimensions

#### 4.2.1 Gamification

The UEREG-CH breaks down “Gamification” into five secondary indicators: challenge, reward, content, plot, and rules, which lacks comprehensive consideration. This study dissects the “Gamification” into “Perceived ease of use,” “Entertainment objective,” and “Pleasure satisfaction,” along with nine tertiary indicators from D21 to D29 (Table 8). It posits that the prerequisite for user engagement in a game is its perceived ease of use, as simple operability leads users to further explore the game’s entertainment aspects. The “Entertainment objective” involves users empathizing with characters, controlling the game’s direction, achieving self-realization, and immersing in the experience, leading to the satisfaction of pleasure and making the game a part of their life. This study views gamification as a distinguishing feature of CH-SGs compared to other educational games, necessitating a multi-faceted consideration of users’ actual operational needs.

**Table 8:** Comparison of indicator dimensions.

**Indicator dimensions**	**UEREG-CH**	**CH-SGs**
Gamification	• Moderate challenge • Reasonable incentives • Optional content • Attractive plot • Clear game rules	Secondary indicators: ‣C8 Perceived ease of use ‣C9 Entertainment objectives ‣C10 Pleasure satisfaction Tertiary indicators: ‣D21—D29
Demand	• Learning content meets or exceeds learner needs • Game functionality meets or exceeds learner need	Secondary indicators: ‣C11 Social satisfaction ‣C12 Achievement satisfaction Tertiary indicators: ‣D30—D34
Social aspect	No	Secondary indicators: ‣C13 Brand image ‣C14 Subjective norms Tertiary indicators: ‣D35—D38
Educational aspect	• Clear and timely knowledge feedback • Level design compliant with learning rules • Reliable content with flexible and varied forms • Clear learning objectives • Balance between skills and challenges	Secondary indicators: ‣C5 Learning objectives ‣C6 Intellectual satisfaction ‣C7 Perceived usefulness Tertiary indicators: ‣D12—D20
Artistic aspect	• Clear interface design • Ordered menu arrangement • Innovative and interesting interface elements • Refined interface production • Pleasant interface feel	Secondary indicators: ‣C1Interface design ‣C2 Immersion satisfaction Tertiary indicators: ‣D1—D6
Usability	• Easy to remember • Easy to operate • Easy to learn • Stable game operation without faults • Clear and timely system feedback	Secondary indicators: ‣C3 System quality ‣C4 Service quality Tertiary indicators: ‣D7—D11

#### 4.2.2 Demand

The UEREG-CH decomposes “Demand” into two aspects: “Game learning content meets or exceeds learner needs” and “Game functionality meets or exceeds learner needs.” This study finds this approach overly simplistic and lacking a clear distinction from “Educational” aspects. CH-SGs categorizes the “Demand dimension” into “Social satisfaction” and “Achievement satisfaction,” with five tertiary indicators from D30 to D34 (Table 8). It believes that users’ intrinsic demands stem from two sources: achievement satisfaction through winning in the game mechanics, and social satisfaction from connections made with other users. These needs are interdependent, as victory in the game leads to a desire for social display and sharing, while collaboration and communication with others facilitate winning.

#### 4.2.3 Social aspect

The UEREG-CH does not set this indicator (Table 8). However, this study argues that games, as products, have strong social attributes, encompassing both users’ social attributes and the game’s societal aspects. CH-SG users often belong to culturally and economically higher echelons, greatly influenced by social norms, and their actions are affected by others in society. If close friends or experts recommend such games, users are likely to try them. The “Brand image” represents the game product’s social attribute. If the game’s production company is well-known, endorsed by celebrities or experts, or developed from a well-known IP, it tends to be favored and sought after by users.

#### 4.2.4 Educational aspect

In the UEREG-CH, “Educational value” is divided into five secondary indicators: “Clear and timely knowledge feedback,” “Levels compliant with learning rules,” “Reliable content with flexible and diverse forms,” “Clear learning objectives,” and “Balanced skills and challenges.” This categorization appears somewhat generic and necessitates further detailed expansion. CH-SGs decompose the “Educational value” into three secondary indicators: “Learning objectives,” “Intellectual satisfaction,” and “Perceived usefulness,” along with nine tertiary indicators from D12 to D20 (Table 8). “Learning objectives” play a crucial role in the quality aspect of CH-SGs games and serve as a distinctive feature differentiating them from other games. Users experience intellectual satisfaction only after completing tasks set by the “Learning objectives.” This sense of intellectual satisfaction, being one of the primary motives for initial game usage, leads to the perception of usefulness, thereby encouraging users to continue using the game. Thus, the relationship among these three indicators is interlinked, each supporting and leading into the next, collectively embodying the educational aspect from multiple perspectives.

#### 4.2.5 Artistic aspect

The UEREG-CH deconstructs “Perceived aesthetics” into interface design, menu, elements, production, etc., which this study believes lacks an overall requirement for the game’s artistry and attention to users’ perception of art. CH-SGs breaks down “Artistic aspect” into two secondary indicators: “Interface design” and “Immersive satisfaction,” along with six tertiary indicators from D1 to D6 (Table 8). This study suggests that the artistic indicators should reflect cultural heritage themes and emphasize creativity more than other educational games. Only when users perceive a unique artistic atmosphere in the interface can they immerse themselves in their game characters and forget the stresses of life.

#### 4.2.6 Usability

The UEREG-CH decomposes “Usability” into indicators such as ease of memorization, operation, learning, absence of faults, and timely feedback. However, these indicators are subjective and semantically vague, overlapping with others. This study breaks down “Usability” into “System quality” and “Service quality,” with five tertiary indicators from D7 to D11 (Table 8). A game’s usability is first judged by system quality; a game is considered usable only when the system is stable, frequently optimized, and has good guidance and synchronous feedback. Additionally, customer service’s timely problem resolution and fulfillment of promises to users are essential; otherwise, users may develop negative perceptions of the game’s usability and abandon it.

### 4.3 Comparative analysis of indicator weights in CH-SGs and UEREG-CH

#### 4.3.1 Weight structure

Both the UEREG-CH and CH-SGs have tree-like indicator systems, but the former does not highlight the characteristics of various serious games as effectively as the latter, which is tailored for CH-SGs. Regarding the method of weight determination, the UEREG-CH uses group AHP cluster analysis, while the CH-SG combines the Delphi method, AHP, and fuzzy comprehensive calculation, blending qualitative and quantitative research (Table 9).

**Table 9:** Comparison of indicator weights.

**Indicator weights**	**UEREG-CH**	**CH-SGs**
Weight structure	• Tree-like indicator systems • Group AHP cluster analysis	• Tree-like indicator systems • Delphi method, AHP, and fuzzy comprehensive calculation
Important indicator weights	• First place “Usability” 0.2547 • Second place “Educational value” 0.2449 • Third place “Needs” 0.2201 • Fourth place “Gamification” 0.1764 • Fifth place “Perceived aesthetics “0.1039	• First place “Gamification” 3.5371 • Second place “Needs” 3.4546 • Third place “Social dimension” 3.4473 • Fourth place “Educational value” 3.4307 • Fifth place “Artistic dimension” 3.4223 • Sixth place “Usability” 3.2355

#### 4.3.2 Important indicator weights

In the UEREG-CH, “Usability” ranks first, followed by “Educational value,” “Demand,” “Gamification,” and “Perceived aesthetics.” This prioritization shows that serious games in China focus more on basic user needs, emphasizing usability and educational aspects but are weaker in gamification and artistic aspects, lacking a balance between learning and gaming. In CH-SGs, “Gamification” ranks first, followed by “Demand,” “Social aspect,” “Educational value,” “Artistic dimension,” and “Usability.” This suggests that CH-SGs users prioritize leisure and entertainment in games, personal needs fulfillment, and social interaction. However, the educational aspect is less emphasized, possibly because CH-SGs, while educational, are perceived as weaker in function compared to pure educational games. This perception might stem from the games’ design issues that fail to emphasize learning objectives through game mechanics, leading to the impression that their educational function is weak. The lesser importance of the artistic aspect could be due to the convergence of artistic styles in current CH-SGs, predominantly adopting traditional Chinese styles and lacking in diversity and innovation. Usability ranks last because system quality generally meets users’ needs in current CH-SGs, and service quality has a minor impact on user experience, thus receiving less attention from users (Table 9).

## 5 Conclusion and limitations

This study confirms that an effective and comprehensive evaluation rubric for CH-SGs can be constructed by focusing on usage motivation, game quality, and continuous usage. Previous research on serious games has focused on the educational aspect and the improvement of learners’ outcomes. However, empirical studies primarily aimed at collecting evidence of learning effectiveness have design flaws, leading to a shift in the research trend beyond just educational aspects. This study observes that the educational function of CH-SGs in China is relatively weak and needs to be integrated into the game mechanics to achieve learning objectives. Compared to other serious games, CH-SGs have a distinct cultural and artistic attribute. Therefore, evaluation rubrics developed for other serious games are not suitable for CH-SGs. To reflect the characteristics of CH-SGs and the needs of their user group, this study distinguishes them from other serious games across six dimensions: “Artistic,” “Usability,” “Educational value,” “Gamification,” “Demand,” and “Social aspect.” The secondary and tertiary indicators emphasize cultural heritage elements, such as breaking down the “Educational value” dimension into technical indicators like “Learning objectives” and non-technical indicators like “Intellectual satisfaction” and “Perceived usefulness,” with tertiary indicators reflecting elements of cultural heritage, such as “Accurate and reliable cultural heritage knowledge in various forms,” “Stimulating curiosity in learning traditional culture,” and “Enhancing the level of traditional cultural knowledge and skills.” Empirical research has revealed a departure from the traditional approach in serious game studies, which often emphasizes a balance between educational and gameplay aspects. It was found that users of CH-SGs tend to favor “Gamification” more, with “Educational” factors ranking only fourth in terms of importance. Therefore, it is recommended that designers of CH-SGs tailor the balance between gaming and learning aspects according to the specific theme and type of each game, rather than uniformly pursuing a balance between the two.

This study also validates that the CH-SGs evaluation rubric is recognized by users, not just experts. Involving users in the evaluation process is crucial, as they have the right to assess the games. Therefore, the evaluation rubric is developed around users’ diverse needs, formed through expert consultations and the AHP method, and further validated by user surveys and fuzzy comprehensive calculations. This approach ensures significant user participation. The study also believes that a game’s true appreciation by users depends on their continued use and the lasting, long-term impact it has on them. Thus, the rubric differs from previous studies by including long-term indicators, focusing on users’ experiences, attitudes, enthusiasm, persistence, and other non-technical aspects (Yu, 2016). Starting from the theory of continued usage intention, the study sets “Perceived usefulness,” “Perceived ease of use,” and “Subjective norms” as secondary indicators, and “Continuous and systematic learning content,” “Long-term contact with other users and feeling care among friends,” etc., as tertiary indicators to assess the long-term impact of CH-SGs on users. True feedback for game iterative improvement can only be obtained through long-term usage by users.

This study also has limitations in its research methodology. Although user needs and evaluations were incorporated in the fuzzy calculation process of establishing the evaluation rubric, there are shortcomings in the composition of the user sample data collected. The survey respondents were all users who had experienced CH-SGs, but many potential users were not surveyed. Additionally, the user groups need further segmentation, as different groups may have varying evaluations of serious games, leading to instability in results. The methods for collecting user needs should also be expanded beyond questionnaires, including in-depth interviews and group discussions for a more detailed understanding of user needs, thus improving the accuracy of the evaluation results. In the Delphi and AHP methods, the study included opinions from an expert panel, which was not fully comprehensive, being predominantly from the field of game studies. This led to subjectivity and bias in the evaluation system and weightings. The inclusion of experts from educational sciences, cultural heritage inheritors, game industry professionals, and other cultural institutions is needed for a more balanced perspective. Moreover, the indicators in the evaluation rubric are not fully comprehensive, with many derived from educational game evaluations. Regarding the design scales for evaluation indicators, this study used a 5-point rating scale, and the scoring of each indicator primarily relied on subjective user perceptions without more detailed criteria. Therefore, how to delineate evaluation levels more finely and completely is a topic for future discussion. Lastly, as the CH-SGs evaluation rubric developed in this study has significant cultural specificity within the serious games domain, whether it can serve as a referable and extendable evaluation paradigm for other types of serious games requires continuous validation in future research.

## Data availability statement

The raw data supporting the conclusions of this article will be made available by the authors, without undue reservation.

## Author contributions

PM: Writing – original draft. DC: Writing – review & editing.
