# Supplementary material for: Research on an evaluation rubric for promoting user’s continuous usage intention: a case study of serious games for Chinese cultural heritage
Source: Front Psychol. 2024 Feb 15;15:1300686. doi: 10.3389/fpsyg.2024.1300686 (PMC10901983; doi:10.3389/fpsyg.2024.1300686)
Supplement: Supplementary file 1 [file Data_Sheet_1.PDF]

Supplementary Material

Research on An Evaluation Rubric for Promoting User's Continuous Usage Intention: A Case Study of Serious Games for Chinese Cultural Heritage

AHP Calculation:

Constructing the Judgment Matrix: Within the comprehensive evaluation system, there are six primary criteria and thirty-eight indicators. Their importance in the comprehensive evaluation varies. Criteria of greater importance should be assigned more significant weights, and vice versa. The weights are determined using the AHP, ultimately resulting in a weight vector for each evaluation indicator. Initially, a judgment matrix at the goal layer is constructed based on the comparative importance of each indicator.

Table 1: Goal Layer Judgment Matrix

Evaluation index	B1 Artistic	B2 Usability	B3 Educational	B4 Gamification	B5 Needs	B6 Social	Wi
B1 Artistic Dimension	1	1	2	1	2	2	0.2255
B2 Usability	1	1	1	1	2	1	0.1789
B3 Educational Value	1/2	1	1	1	2	1/2	0.1420
B4 Gamification	1	1	1	1	3	3	0.2299
B5 Needs	1/2	1/2	1/2	1/3	1	2	0.1054
B6 Social Dimension	1/2	1	2	1/3	1/2	1	0.1183

From Table 1,**λmax=6.4380**，CI=0.0876，RI=1.24,CR=0.0707，CR<0.1，the matrix exhibits satisfactory consistency.

The detailed calculation process for the matrix weights is as follows:

First, calculate the product of the elements in each row of the judgment matrix，$m_i = \prod_{j=1} a_{ij}$ **=[8.0000,2.0000,0.5000,9.0000,0.0833,0.1667]**

Then, calculate the nth root of mi，$w_i^* = \sqrt[n]{m_i}$ = [1.4142, 1.1225, 0.8909, 1.4422, 0.6609, 0.7418]。

Next, normalize the vector：$w_i = w_i^* / \sum_{i=1}^{n} w_i^*$ = [0.2255, 0.1789, 0.1420, 0.2299, 0.1054, 0.1183]

The calculation for the largest eigenvalueλ_{max} is: $\lambda_{max} = \frac{1}{n}\sum_{i=1}^{n}\frac{(Aw)i}{w_i}$=1/6 × 38.6282=6.4380

Which,
Aw_i=[1.3657,1.1054,0.9335,1.4473,0.6918,0.8233]。

The Consistency Index CI is determined as: $CI = \frac{\lambda_{max} - n}{n-1}$=(6.4380-6)/(6-1)=0.0876

From the RI table, when the judgment matrix is of order 6, RI is 1.24.
The Average Consistency CR is calculated as: CR=CI/RI=0.0876/1.24=0.0707<0.1, passing the consistency test.

Table 2: Judgment Matrix

Evaluation index	C1 Interface Design	C2 Immersion Satisfaction	Wi
C1 Interface Design	1	2	0.6667
C2 Immersion Satisfaction	1/2	1	0.3333

From Table 2, λ_{max}=2.0000, CI=0.0000, RI=0, CR=0.0000, The matrix exhibits satisfactory consistency.

It is evident from the table that the importance of C1 (Interface Design) is twice that of C2 (Immersion Satisfaction). Based on the fundamental concept of the judgment matrix, the weights of the two can be determined.

Table 3: Judgment Matrix

Evaluation index	C3 System Quality	C4 Service Quality	Wi
C3 System Quality	1	2	0.6667
C4 Service Quality	1/2	1	0.3333

From Table 3, λ_{max}=2.0000, CI=0.0000, RI=0, CR=0.0000. The matrix exhibits satisfactory consistency.

As indicated in the table, the importance of C3 (System Quality) is twice that of C4 (Service Quality). Based on the fundamental concept of the judgment matrix, the weights of the two can be determined.

Table 4: Judgment Matrix

Evaluation index	C5 Learning Objectives	C6 Intellectual Satisfaction	C7 Perceived Usefulness	Wi
C5 Learning Objectives	1	2	2	0.4934
C6 Intellectual Satisfaction	1/2	1	1/2	0.1958
C7 Perceived Usefulness	1/2	2	1	0.3108

From Table 4, λ_{max}=3.0536, CI=0.0268, RI=0.58,CR=0.0462, CR<0.1. The matrix exhibits satisfactory consistency.

The detailed calculation process for the matrix weights is as follows:
First, calculate the product of the elements in each row of the judgment matrix,

$m_i = \prod_{j=1} a_{ij}$ =[4.0000,0.2500,1.0000]^T。

Then, calculate the nth root of mi, $w_i^* = \sqrt[n]{m_i}$ = [1.5874, 0.6300, 1.0000]。

Next, normalize the vector： $w_i = w_i^* / \sum_{i=1}^{n} w_i^*$ = [0.4934, 0.1958, 0.3108]

The calculation for the largest eigenvalue λ_{max} is： $\lambda_{max} = \frac{1}{n}\sum_{i=1}^{n}\frac{(Aw)i}{w_i}$=1/3 × 9.1609=3.0536

Which, Aw_i=[1.5066,0.5979,0.9491].

The Consistency Index CI is determined as： $CI = \frac{\lambda_{max} - n}{n-1}$ =(3.0536-3)/(3-1)=0.0268

From the RI table, when the judgment matrix is of order 3, RI is 0.58.
The Average Consistency CR is calculated as：CR=CI/RI=0.0268/0.58=0.0462<0.1, passing the consistency test.

Table 5: Judgment Matrix

Evaluation index	C8 Perceived Ease of Use	C9 Entertainment Objectives	C10 Pleasure Satisfaction	Wi
C8 Perceived Ease of Use	1	2	2	0.5000
C9 Entertainment Objectives	1/2	1	1	0.2500
C10 Pleasure Satisfaction	1/2	1	1	0.2500

From Table 6, λ_{max}=3.0000， CI=0.0000， RI=0.58,CR=0.0000， CR<0.1. The matrix exhibits satisfactory consistency.
The detailed calculation process for the matrix weights is as follows:
First, calculate the product of the elements in each row of the judgment matrix,

$m_i = \prod_{j=1} a_{ij}$ =[4.0000,0.5000,0.5000]^T。

Then, calculate the nth root of mi, $w_i^* = \sqrt[n]{m_i}$ = [1.5874, 0.7937, 0.7937].

Next, normalize the vector: $w_i = w_i^* / \sum_{i=1}^{n} w_i^*$ = [0.5000, 0.2500, 0.2500].

The calculation for the largest eigenvalue λ_{max} is： $\lambda_{max} = \frac{1}{n}\sum_{i=1}^{n}\frac{(Aw)i}{w_i}$=1/3 × 9.0000=3.0000

Which, Aw_i=[1.5000,0.7500,0.7500]。

The Consistency Index CI is determined as: $CI = \frac{\lambda_{max} - n}{n-1}$ =(3.0000-3)/(3-1)=0.0000

The Average Consistency CR is calculated as：CR=CI/RI=0.0000/0.58=0.0000<0.1, passing the

consistency test.

Table 6: Judgment Matrix

Evaluation index	C11Social Satisfaction	C12Achievement Satisfaction	Wi
C11 Social Satisfaction	1	1	0.5000
C12Achievement Satisfaction	1	1	0.5000

From Table 6, λ_{max}=2.0000， CI=0.0000， RI=0， CR=0.0000. The matrix exhibits satisfactory consistency.

As indicated in the table, the importance of C11 (Social Satisfaction) is twice that of C12 (Achievement Satisfaction). Based on the fundamental concept of the judgment matrix, the weights of the two can be determined.

Table 7: Judgment Matrix

Evaluation index	C13 Brand Image	C14 Subjective Norms	Wi
C13 Brand Image	1	2	0.6667
C14 Subjective Norms	1/2	1	0.3333

From Table 7, λ_{max}=2.0000， CI=0.0000， RI=0， CR=0.0000. The matrix exhibits satisfactory consistency.

As indicated in the table, the importance of C13 (Brand Image) is twice that of C14 (Subjective Norms). Based on the fundamental concept of the judgment matrix, the weights of the two can be determined.

Table 8: Judgment Matrix

Evaluation index	D1 Interface design features cultural heritage theme	D2 Interface menu layout is reasonable and orderly	D3 Creative character roles and cultural skill settings	D4 High Clarity, Exquisite Realism, and Interesting Visual Scenes	Wi
D1 Interface design features cultural heritage theme	1	1	2	1	0.2870
D2 Interface menu layout is reasonable and orderly	1	1	1	1	0.2413
D3 Creative character roles and cultural skill settings.	1/2	1	1	1/3	0.1542
D4 High Clarity, Exquisite Realism, and Interesting Visual Scenes	1	1	3	1	0.3176

From Table 8, λ_{max}=4.1171， CI=0.0390， RI=0.9,CR=0.0434， CR<0.1, The matrix exhibits satisfactory consistency.

The detailed calculation process for the matrix weights is as follows:

First, calculate the product of the elements in each row of the judgment matrix,

$m_i = \prod_{j=1} a_{ij}$ =[2.0000,1.0000,0.1667,3.0000]

Then, calculate the nth root of mi, $w_i^* = \sqrt[n]{m_i}$ = [1.1892, 1.0000, 0.6389, 1.3161]。

Next, normalize the vector: $w_i = w_i^* / \sum_{i=1}^{n} w_i^*$ = [0.2870, 0.2413, 0.1542, 0.3176]

The calculation for the largest eigenvalue λ_{max} is： $\lambda_{max} = \frac{1}{n} \sum_{i=1}^{n} \frac{(Aw)i}{w_i}$ =1/4× 16.4686=4.1171

Which, Aw_i=[1.1542,1.0000,0.6448,1.3084]。

The Consistency Index CI is determined as: $CI = \frac{\lambda_{max} - n}{n-1}$ =(4.1171-4)/(4-1)=0.0390

The Average Consistency CR is calculated as： CR=CI/RI=0.0390/0.9=0.0434<0.1, passing the consistency test.

Table 9: Judgment Matrix

Evaluation index	D5 Immersing in creating one's story with artistic roles	D6 Helping to forget the problems and stresses of real life	Wi
D5 Immersing in creating one's story with artistic roles	1	3	0.7500
D6 Helping to forget the problems and stresses of real life	1/3	1	0.2500

From Table 9, λ_{max}=2.0000， CI=0.0000， RI=0， CR=0.0000. The matrix exhibits satisfactory consistency.

As indicated in the table, the importance of D5 Immersing in creating one's story with artistic roles is triple that of D6 Helping to forget the problems and stresses of real life. Based on the fundamental concept of the judgment matrix, the weights of the two can be determined.

Table 10: Judgment Matrix

Evaluation index	D7 Stable game system, bug-free, regular updates, and optimization	D8 Effective game guidance, helping users access necessary game information	D9 Synchronous feedback mode is diverse, timely, and recognizable	Wi
D7 Stable game system, bug-free, regular updates, and optimization	1	2	2	0.4934
D8 Effective game guidance, helping users access necessary game	1/2	1	1/2	0.1958

information				
D9 Synchronous feedback mode is diverse, timely, and recognizable	1/2	2	1	0.3108

From Table 10, λ_{max}=3.0536， CI=0.0268， RI=0.58,CR=0.0462， CR<0.1, The matrix exhibits satisfactory consistency.

The detailed calculation process for the matrix weights is as follows:

First, calculate the product of the elements in each row of the judgment matrix,

$m_i = \prod_{j=1} a_{ij}$ =[4.0000,0.2500,1.0000]^T。

Then, calculate the nth root of mi, $w_i^* = \sqrt[n]{m_i}$ = [1.5874, 0.6300, 1.0000]。

Next, normalize the vector: $w_i = w_i^* / \sum_{i=1}^{n} w_i^*$ = [0.4934, 0.1958, 0.3108]

The calculation for the largest eigenvalue λ_{max} is： $\lambda_{max} = \frac{1}{n}\sum_{i=1}^{n}\frac{(Aw)i}{w_i}$=1/3 × 9.1609=3.0536

Which, Aw_i=[1.5066,0.5979,0.9491]。

The Consistency Index CI is determined as: $CI = \frac{\lambda_{max} - n}{n-1}$ =(3.0536-3)/(3-1)=0.0268

From the RI table, when the judgment matrix is of order 3, RI is 0.58.

The Average Consistency CR is calculated as: CR=CI/RI=0.0268/0.58=0.0462<0.1, passing the consistency test.

Table11: Judgment Matrix

Evaluation index	D10 Regular promotional activities by the game manufacturer and fulfillment of promises to users	D11 Customer Service Staff Being Competent and Promptly Resolving User Issues	Wi
D10 Regular promotional activities by the game manufacturer and fulfillment of promises to users	1	2	0.6667
D11 Customer Service Staff Being Competent and Promptly Resolving User Issues	1/2	1	0.3333

From Table 11, λ_{max}=2.0000， CI=0.0000， RI=0， CR=0.0000. The matrix exhibits satisfactory consistency.

As indicated in the table, the importance of D10 Regular promotional activities by the game manufacturer and fulfillment of promises to users is twice that of D11 Customer Service Staff Being Competent and Promptly Resolving User Issues. Based on the fundamental concept of the

judgment matrix, the weights of the two can be determined.

Table 12: Judgment Matrix

Evaluation index	D12 The learning content is continuous and systematic	D13 The learning objectives are clear, rigorous, and not distracted by games	D14 Knowledge feedback is positive and multi-formally rewarding	D15 Cultural heritage knowledge is accurate, reliable, and varied in form	D16 Level design conforms to learning laws and user cognitive levels	Wi
D12 The learning content is continuous and systematic	1	2	2	2	2	0.3303
D13 The learning objectives are clear, rigorous, and not distracted by games	1/2	1	2	1	1/2	0.1651
D14 Knowledge feedback is positive and multi-formally rewarding	1/2	1/2	1	1	1/2	0.1252
D15 Cultural heritage knowledge is accurate, reliable, and varied in form	1/2	1	1	1	2	0.1897
D16 Level design conforms to learning laws and user cognitive levels	1/2	2	2	1/2	1	0.1897

From Table 12, λ_{max}=5.2758， CI=0.0689， RI=1.12,CR=0.0616， CR<0.1, The matrix exhibits satisfactory consistency.

The detailed calculation process for the matrix weights is as follows:

First, calculate the product of the elements in each row of the judgment matrix,

$m_i = \prod_{j=1} a_{ij}$ =[16.0000,0.5000,0.1250,1.0000,1.0000]

Then, calculate the nth root of mi, $w_i^* = \sqrt[n]{m_i}$ = [1.7411, 0.8706, 0.6598, 1.0000, 1.0000]。

Next, normalize the vector: $w_i = w_i^* / \sum_{i=1}^{n} w_i^*$ = [0.3303, 0.1651, 0.1252, 0.1897, 0.1897]

The calculation for the largest eigenvalue λ_{max} is： $\lambda_{max} = \frac{1}{n}\sum_{i=1}^{n}\frac{(Aw)i}{w_i}$ =1/5 × 26.3789=5.2758

Which, Aw_i=[1.6697,0.8652,0.6574,1.0246,1.0303]。

The Consistency Index CI is determined as: $CI = \frac{\lambda_{max} - n}{n-1}$ =(5.2758-5)/(5-1)=0.0689

From the RI table, when the judgment matrix is of order 5, RI is 1.12.
The Average Consistency CR is calculated as: CR=CI/RI=0.0689/1.12=0.0616<0.1, passing the consistency test.
Table13: Judgment Matrix

Evaluation index	D17 Enhancing the ability to complete tasks and cooperate with others intellectually	D18 Stimulating curiosity to learn traditional culture	Wi
D17 Enhancing the ability to complete tasks and cooperate with others intellectually	1	2	0.6667
D18 Stimulating curiosity to learn traditional culture	1/2	1	0.3333

From Table 13, λ_{max}=2.0000， CI=0.0000， RI=0， CR=0.0000. The matrix exhibits satisfactory consistency.
As indicated in the table, D17 Enhancing the ability to complete tasks and cooperate with others intellectually
is twice that of D18 Stimulating curiosity to learn traditional culture. Based on the fundamental concept of the judgment matrix, the weights of the two can be determined.
Table 14: Judgment Matrix

Evaluation index	D19 Helping in life and study, relieving mental stress	D20 Enhancing the level of traditional cultural knowledge and skills	Wi
D19 Helping in life and study, relieving mental stress	1	2	0.6667
D20 Enhancing the level of traditional cultural knowledge and skills	1/2	1	0.3333

From Table 14, λ_{max}=2.0000， CI=0.0000， RI=0， CR=0.0000. The matrix exhibits satisfactory consistency.
As indicated in the table, D19 Helping in life and study, relieving mental stress is twice that of D20 Enhancing the level of traditional cultural knowledge and skills. Based on the fundamental concept of the judgment matrix, the weights of the two can be determined.
Table 15: Judgment Matrix

Evaluation index	D21 Ease of downloading, installing, and paying for the game	D22 Ease of learning and mastering the game operations	Wi
D21 Ease of downloading, installing, and paying for the game	1	2	0.6667

D22 Ease of learning and mastering the game operations	1/2	1	0.3333

From Table 15, λ_{max}=2.0000， CI=0.0000， RI=0， CR=0.0000. The matrix exhibits satisfactory consistency.

As indicated in the table, D21 Ease of downloading, installing, and paying for the game is twice that of D22 Ease of learning and mastering the game operations. Based on the fundamental concept of the judgment matrix, the weights of the two can be determined.

Table 16: Judgment Matrix

Evaluation index	D23 Establishing multi-modal experiences such as graphics, sound effects, and touch	D24 Able to highly concentrate attention and empathize with characters	D25 Can control to affect game difficulty or plot direction	D26 Realizing self-creation, self-expression, exploration, and discovery	D27 Obtaining pleasure, mental enjoyment, relaxation, and immersion	Wi
D23 Establishing multi-modal experiences such as graphics, sound effects, and touch	1	3	2	2	2	0.3426
D24 Able to highly concentrate attention and empathize with characters	1/3	1	1/2	1/3	1/2	0.0886
D25 Can control to affect game difficulty or plot direction	1/2	2	1	1	1/2	0.1580
D26 Realizing self-creation, self-expression, exploration, and discovery	1/2	3	1	1	1/2	0.1713
D27 Obtaining pleasure, mental enjoyment, relaxation, and immersion	1/2	2	2	2	1	0.2394

From Table 16, λ_{max}=5.1398， CI=0.0349， RI=1.12,CR=0.0312， CR<0.1, The matrix exhibits satisfactory consistency.

The detailed calculation process for the matrix weights is as follows:

First, calculate the product of the elements in each row of the judgment matrix,

$m_i = \prod_{j=1} a_{ij}$ =[24.0000,0.0278,0.5000,0.7500,4.0000]

Then, calculate the nth root of mi, $w_i^* = \sqrt[n]{m_i}$ = [1.8882, 0.4884, 0.8706, 0.9441, 1.3195]。

Next, normalize the vector: $w_i = w_i^* / \sum_{i=1}^{n} w_i^*$ = [0.3426, 0.0886, 0.1580, 0.1713, 0.2394]

The calculation for the largest eigenvalue λmax is： $\lambda_{max} = \frac{1}{n}\sum_{i=1}^{n}\frac{(Aw)i}{w_i}$ =1/5 × 25.6988=5.1398

Which, Aw_i=[1.7460,0.4587,0.7976,0.8862,1.2466]。

The Consistency Index CI is determined as:： $CI = \frac{\lambda_{max} - n}{n-1}$ =(5.1398-5)/(5-1)=0.0349

From the RI table, when the judgment matrix is of order 5, RI is 1.12.
The Average Consistency CR is calculated as: CR=CI/RI=0.0349/1.12=0.0312<0.1, passing the consistency test.

Table17: Judgment Matrix

Evaluation index	D28 Enriching fragmented time in life, increasing topics for conversation	D29 Participating in offline experiences and purchase of traditional culture-related products	Wi
D28 Enriching fragmented time in life, increasing topics for conversation	1	2	0.6667
D29 Participating in offline experiences and purchase of traditional culture-related products	1/2	1	0.3333

From Table 17, λ_{max}=2.0000， CI=0.0000， RI=0， CR=0.0000. The matrix exhibits satisfactory consistency.

As indicated in the table, D28 Enriching fragmented time in life, increasing topics for conversation is twice that of D29 Participating in offline experiences and purchase of traditional culture-related products. Based on the fundamental concept of the judgment matrix, the weights of the two can be determined.

Table 18: Judgment Matrix

Evaluation index	D30 Can choose games with different modes like interaction, competition, collaboration with others	D31 Making Many Friends through Cooperation and Communication with Other Users	D32 Long-term Contact with Other Users and Feeling Care Among Friends	Wi
D30 Can choose games with different modes like	1	2	2	0.4934

interaction, competition, collaboration with others				
D31 Making Many Friends through Cooperation and Communication with Other Users	1/2	1	2	0.3108
D32 Long-term Contact with Other Users and Feeling Care Among Friends	1/2	1/2	1	0.1958

From Table 18, λ_{max}=3.0536, CI=0.0268, RI=0.58,CR=0.0462, CR<0.1. The matrix exhibits satisfactory consistency.

The detailed calculation process for the matrix weights is as follows:

First, calculate the product of the elements in each row of the judgment matrix,

$m_i = \prod_{j=1} a_{ij}$ =[4.0000,1.0000,0.2500]^T。

Then, calculate the nth root of mi, $w_i^* = \sqrt[n]{m_i}$ = [1.5874, 1.0000, 0.6300]。

Next, normalize the vector: $w_i = w_i^* / \sum_{i=1}^{n} w_i^*$ = [0.4934, 0.3108, 0.1958]

The calculation for the largest eigenvalue λ_{max} is： $\lambda_{max} = \frac{1}{n}\sum_{i=1}^{n}\frac{(Aw)i}{w_i}$ =1/3 × 9.1609=3.0536

Which, Aw_i=[1.5066,0.9491,0.5979]。

The Consistency Index CI is determined as: $CI = \frac{\lambda_{max} - n}{n-1}$ =(3.0536-3)/(3-1)=0.0268

From the RI table, when the judgment matrix is of order 3, RI is 0.58.

The Average Consistency CR is calculated as: CR=CI/RI=0.0268/0.58=0.0462<0.1, passing the consistency test.

Table19: Judgment Matrix

Evaluation index	D33 The Desire to Win in Challenges and Competitions	D34 Rapid progression of the game character by mastering the rules, achieving wealth and status in the game	Wi
D33 The Desire to Win in Challenges and Competitions	1	2	0.6667
D34 Rapid progression of the game character by mastering the rules, achieving wealth and status in the game	1/2	1	0.3333

From Table 19, λ_{max}=2.0000， CI=0.0000， RI=0， CR=0.0000. The matrix exhibits satisfactory consistency.

As indicated in the table, D33 The Desire to Win in Challenges and Competitions is twice that of D34 Rapid progression of the game character by mastering the rules, achieving wealth and status in the game. Based on the fundamental concept of the judgment matrix, the weights of the two can be determined.

Table 20: Judgment Matrix

Evaluation index	D35 High Recognition or Liked Cultural IP Themes	D36 Production Companies with High Recognition or Liked Experts and Stars	Wi
D35 High Recognition or Liked Cultural IP Themes	1	2	0.6667
D36 Production Companies with High Recognition or Liked Experts and Stars	1/2	1	0.3333

From Table20, λ_{max}=2.0000， CI=0.0000， RI=0， CR=0.0000. The matrix exhibits satisfactory consistency.

As indicated in the table, D35 High Recognition or Liked Cultural IP Themes is twice that of D36 Production Companies with High Recognition or Liked Experts and Stars. Based on the fundamental concept of the judgment matrix, the weights of the two can be determined.

Table 21: Judgment Matrix

Evaluation index	D37 Many people around me play this game and recommend it to me	D38 Recommendations for the game by experts, media, or important people in my life	Wi
D37 Many people around me play this game and recommend it to me	1	3	0.7500
D38 Recommendations for the game by experts, media, or important people in my life	1/3	1	0.2500

From Table21, λ_{max}=2.0000， CI=0.0000， RI=0， CR=0.0000. The matrix exhibits satisfactory consistency.

As indicated in the table, D37 Many people around me play this game and recommend it to me is twice that of D38 Recommendations for the game by experts, media, or important people in my life. Based on the fundamental concept of the judgment matrix, the weights of the two can be determined.

Table 22: Judgment Matrix

Evaluation index	Wi	λmax	CI	RI	CR
B1	0.2255	6.4380	0.0876	1.24	0.0707
B2	0.1789				
B3	0.1420				
B4	0.2299				
B5	0.1054				
B6	0.1183				
C1	0.6667	2.0000	0.0000	0	0.0000
C2	0.3333				

C3	0.6667	2.0000	0.0000	0	0.0000
C4	0.3333				
C5	0.4934	3.0536	0.0268	0.58	0.0462
C6	0.1958				
C7	0.3108				
C8	0.5000	3.0000	0.0000	0.58	0.0000
C9	0.2500				
C10	0.2500				
C11	0.5000	2.0000	0.0000	0	0.0000
C12	0.5000				
C13	0.6667	2.0000	0.0000	0	0.0000
C14	0.3333				
D1	0.2870	4.1171	0.0390	0.9	0.0434
D2	0.2413				
D3	0.1542				
D4	0.3176				
D5	0.7500	2.0000	0.0000	0	0.0000
D6	0.2500				
D7	0.4934	3.0536	0.0268	0.58	0.0462
D8	0.1958				
D9	0.3108				
D10	0.6667	2.0000	0.0000	0	0.0000
D11	0.3333				
D12	0.3303	5.2758	0.0689	1.12	0.0616
D13	0.1651				
D14	0.1252				
D15	0.1897				
D16	0.1897				
D17	0.6667	2.0000	0.0000	0	0.0000
D18	0.3333				
D19	0.6667	2.0000	0.0000	0	0.0000
D20	0.3333				
D21	0.6667	2.0000	0.0000	0	0.0000
D22	0.3333				
D23	0.3426	5.1398	0.0349	1.12	0.0312
D24	0.0886				
D25	0.1580				
D26	0.1713				
D27	0.2394				
D28	0.6667	2.0000	0.0000	0	0.0000
D29	0.3333				
D30	0.4934	3.0536	0.0268	0.58	0.0462
D31	0.3108				
D32	0.1958				
D33	0.6667	2.0000	0.0000	0	0.0000
D34	0.3333				

D35	0.6667	2.0000	0.0000	0	0.0000
D36	0.3333				
D37	0.7500	2.0000	0.0000	0	0.0000
D38	0.2500				
Note: When CR is less than 0.1, it is considered that the consistency of the judgment matrix is acceptable					

User Questionnaire for Fuzzy Comprehensive Evaluation

Question Item	1-Poor	2-Fair	3-Average	4-Good	5-Excellent
D1 Interface design features cultural heritage theme					
D2 Interface menu layout is reasonable and orderly					
D3 Creative character roles and cultural skill settings.					
D4 High Clarity, Exquisite Realism, and Interesting Visual Scenes					
D5 Immersing in creating one's story with artistic roles					
D6 Helping to forget the problems and stresses of real life					
D7 Stable game system, bug-free, regular updates, and optimization					
D8 Effective game guidance, helping users access necessary game information					
D9 Synchronous feedback mode is diverse, timely, and recognizable					
D10 Regular promotional activities by the game manufacturer and fulfillment of promises to users					
D11 Customer Service Staff Being Competent and Promptly Resolving User Issues					
D12 The learning content is continuous and systematic					
D13 The learning objectives are clear, rigorous, and not distracted by games					
D14 Knowledge feedback is positive and multi-formally rewarding					
D15 Cultural heritage knowledge is accurate, reliable, and varied in form					
D16 Level design conforms to learning laws and user cognitive levels					
D17 Enhancing the ability to complete tasks and cooperate with others intellectually					
D18 Stimulating curiosity to learn traditional culture					
D19 Helping in life and study, relieving mental stress					
D20 Enhancing the level of traditional cultural					

knowledge and skills					
D21 Ease of downloading, installing, and paying for the game					
D22 Ease of learning and mastering the game operations					
D23 Establishing multi-modal experiences such as graphics, sound effects, and touch					
D24 Able to highly concentrate attention and empathize with characters					
D25 Can control to affect game difficulty or plot direction					
D26 Realizing self-creation, self-expression, exploration, and discovery					
D27 Obtaining pleasure, mental enjoyment, relaxation, and immersion					
D28 Enriching fragmented time in life, increasing topics for conversation					
D29 Participating in offline experiences and purchase of traditional culture-related products					
D30 Can choose games with different modes like interaction, competition, collaboration with others					
D31 Making Many Friends through Cooperation and Communication with Other Users					
D32 Long-term Contact with Other Users and Feeling Care Among Friends					
D33 The Desire to Win in Challenges and Competitions					
D34 Rapid progression of the game character by mastering the rules, achieving wealth and status in the game					
D35 High Recognition or Liked Cultural IP Themes					
D36 Production Companies with High Recognition or Liked Experts and Stars					
D37 Many people around me play this game and recommend it to me					
D38 Recommendations for the game by experts, media, or important people in my life					

Fuzzy Comprehensive Evaluation

Fuzzy Element Hierarchical Structure

First, present the indicators for each level

U={ B1 Artistic Dimension, B2 Usability, B3 Educational Value, B4 Gamification, B5 Needs, B6

Social Dimension}
UB1 Artistic Dimension ={ C1Interface Design, C2 Immersion Satisfaction }，
UB2 Usability ={C3 System Quality, C4 Service Quality}，
UB3 Educational Value ={C5 Learning Objectives, C6 Intellectual Satisfaction, C7 Perceived Usefulness}，
UB4 Gamification ={C8 Perceived Ease of Use, C9 Entertainment Objectives, C10 Pleasure Satisfaction}，
UB5 Needs ={C11 Social Satisfaction, C12 Achievement Satisfaction}，
UB6 Social Dimension ={C13 Brand Image, C14 Subjective Norms}。
UC1Interface Desig={ D1 Interface design features cultural heritage theme, D2 Interface menu layout is reasonable and orderly, D3 Creative character roles and cultural skill settings, D4 High Clarity, Exquisite Realism, and Interesting Visual Scenes},
UC2 Immersion Satisfaction ={ D5 Immersing in creating one's story with artistic roles, D6 Helping to forget the problems and stresses of real life },
UC3 System Quality ={ D7 Stable game system, bug-free, regular updates, and optimization, D8 Effective game guidance, helping users access necessary game information, D9 Synchronous feedback mode is diverse, timely, and recognizable},
UC4 Service Quality ={ D10 Regular promotional activities by the game manufacturer and fulfillment of promises to users, D11 Customer Service Staff Being Competent and Promptly Resolving User Issues},
UC5 Learning Objectives ={ D12 The learning content is continuous and systematic , D13 The learning objectives are clear, rigorous, and not distracted by games, D14 Knowledge feedback is positive and multi-formally rewarding, D15 Cultural heritage knowledge is accurate, reliable, and varied in form, D16 Level design conforms to learning laws and user cognitive levels},
UC6 Intellectual Satisfaction ={ D17 Enhancing the ability to complete tasks and cooperate with others intellectually, D18 Stimulating curiosity to learn traditional culture},
UC7 Perceived Usefulness ={ D19 Helping in life and study, relieving mental stress, D20 Enhancing the level of traditional cultural knowledge and skills},
UC8 Perceived Ease of Use ={ D21 Ease of downloading, installing, and paying for the gameD22 Ease of learning and mastering the game operations },
UC9 Entertainment Objectives ={D23 Establishing multi-modal experiences such as graphics, sound effects, and touch, D24 Able to highly concentrate attention and empathize with characters, D25 Can control to affect game difficulty or plot direction, D26 Realizing self-creation, self-expression, exploration, and discovery, D27 Obtaining pleasure, mental enjoyment, relaxation, and immersion},
UC10 Pleasure Satisfaction ={D28 Enriching fragmented time in life, increasing topics for conversation, D29 Participating in offline experiences and purchase of traditional culture-related products},
UC11 Social Satisfaction ={D30 Can choose games with different modes like interaction, competition, collaboration with others, D31 Making Many Friends through Cooperation and Communication with Other Users, D32 Long-term Contact with Other Users and Feeling Care Among Friends},
UC12 Achievement Satisfaction ={D33 The Desire to Win in Challenges and Competitions, D34 Rapid progression of the game character by mastering the rules, achieving wealth and status in the

game},
UC13 Brand Image ={D35 High Recognition or Liked Cultural IP Themes, D36 Production Companies with High Recognition or Liked Experts and Stars},
UC14 Subjective Norms ={D37 Many people around me play this game and recommend it to me, D38 Recommendations for the game by experts, media, or important people in my life}.
Fuzzy Element Weights and Evaluation Set Scores
First, present the indicators for each level
W={0.2255,0.1789,0.1420,0.2299,0.1054,0.1183}。
WB1={0.6667,0.3333}，
WB2={0.6667,0.3333}，
WB3={0.4934,0.1958,0.3108}，
WB4={0.5000,0.2500,0.2500}，
WB5={0.5000,0.5000}，
WB6={0.6667,0.3333}。
WC1={0.2870,0.2413,0.1542,0.3176},
WC2={0.7500,0.2500},
WC3={0.4934,0.1958,0.3108},
WC4={0.6667,0.3333},
WC5={0.3303,0.1651,0.1252,0.1897,0.1897},
WC6={0.6667,0.3333},
WC7={0.6667,0.3333},
WC8={0.6667,0.3333},
WC9={0.3426,0.0886,0.1580,0.1713,0.2394},
WC10={0.6667,0.3333},
WC11={0.4934,0.3108,0.1958},
WC12={0.6667,0.3333},
WC13={0.6667,0.3333},
WC14={0.7500,0.2500}。
Provide the evaluation set and corresponding scores as: Y = {Poor, Fair, Average, Good, Excellent} = {1, 2, 3, 4, 5}.
Fuzzy Element Weights and Evaluation Set Scores
First-Level Fuzzy Comprehensive Evaluation:
The fuzzy matrix (membership degree matrix) corresponding to C1 is obtained as: Table 1.

C1	Poor	Fair	Average	Good	Excellent
D1	0.0332	0.1301	0.4592	0.2474	0.1301
D2	0.1148	0.1760	0.3010	0.2704	0.1378
D3	0.1454	0.2985	0.3571	0.0816	0.1173
D4	0.0969	0.1276	0.1684	0.2219	0.3852

The fuzzy matrix (membership degree matrix) corresponding to C2 is obtained as: Table2

C2	Poor	Fair	Average	Good	Excellent
D5	0.0944	0.1071	0.1709	0.3214	0.3061
D6	0.0689	0.0969	0.1658	0.3010	0.3673

The fuzzy matrix (membership degree matrix) corresponding to C3 is obtained as: Table3

C3	Poor	Fair	Average	Good	Excellent
D7	0.0561	0.1505	0.4388	0.2372	0.1173
D8	0.1735	0.3036	0.3138	0.0995	0.1097
D9	0.1020	0.1301	0.3929	0.2066	0.1684

The fuzzy matrix (membership degree matrix) corresponding to C4 is obtained as: Table4

C4	Poor	Fair	Average	Good	Excellent
D10	0.0893	0.0842	0.2934	0.3291	0.2041
D11	0.0536	0.1199	0.2679	0.3393	0.2194

The fuzzy matrix (membership degree matrix) corresponding to C5 is obtained as: Table5

C5	Poor	Fair	Average	Good	Excellent
D12	0.0689	0.1122	0.1480	0.3010	0.3699
D13	0.0893	0.1250	0.4184	0.2168	0.1505
D14	0.0765	0.1633	0.2143	0.3776	0.1684
D15	0.0816	0.0663	0.1939	0.2551	0.4031
D16	0.0714	0.1327	0.2526	0.3699	0.1735

The fuzzy matrix (membership degree matrix) corresponding to C6 is obtained as: Table6

C6	Poor	Fair	Average	Good	Excellent
D17	0.0561	0.1122	0.2321	0.2806	0.3189
D18	0.0587	0.1276	0.2015	0.2806	0.3316

The fuzzy matrix (membership degree matrix) corresponding to C7 is obtained as: Table7

C7	Poor	Fair	Average	Good	Excellent
D19	0.0893	0.2117	0.3189	0.2449	0.1352
D20	0.1556	0.2883	0.3061	0.1071	0.1429

The fuzzy matrix (membership degree matrix) corresponding to C8 is obtained as: Table8

C8	Poor	Fair	Average	Good	Excellent
D21	0.0816	0.1378	0.2041	0.2219	0.3546
D22	0.0944	0.1276	0.1735	0.3214	0.2832

The fuzzy matrix (membership degree matrix) corresponding to C9 is obtained as: Table9

C9	Poor	Fair	Average	Good	Excellent
D23	0.0867	0.0842	0.1735	0.2653	0.3903
D24	0.0969	0.1403	0.4005	0.2347	0.1276
D25	0.1556	0.3163	0.2883	0.1148	0.1250
D26	0.0842	0.1429	0.4056	0.2347	0.1327
D27	0.0995	0.0765	0.3240	0.3010	0.1990

The fuzzy matrix (membership degree matrix) corresponding to C10 is obtained as: Table10

C10	Poor	Fair	Average	Good	Excellent
D28	0.0536	0.1582	0.2500	0.3163	0.2219
D29	0.0969	0.1199	0.1505	0.2883	0.3444

The fuzzy matrix (membership degree matrix) corresponding to C11 is obtained as: Table11

C11	Poor	Fair	Average	Good	Excellent

D30	0.0765	0.1556	0.4031	0.1888	0.1760
D31	0.0740	0.1327	0.2092	0.4133	0.1709
D32	0.0485	0.0714	0.2194	0.2577	0.4031

The fuzzy matrix (membership degree matrix) corresponding to C12 is obtained as: Table12

C12	Poor	Fair	Average	Good	Excellent
D33	0.0663	0.1352	0.2066	0.3724	0.2194
D34	0.1378	0.1173	0.2372	0.2934	0.2143

The fuzzy matrix (membership degree matrix) corresponding to C13 is obtained as: Table13

C13	Poor	Fair	Average	Good	Excellent
D35	0.0663	0.1148	0.3061	0.3291	0.1837
D36	0.0740	0.1020	0.1735	0.3036	0.3469

The fuzzy matrix (membership degree matrix) corresponding to C14 is obtained as: Table14

C14	Poor	Fair	Average	Good	Excellent
D37	0.0969	0.1250	0.4362	0.1786	0.1633
D38	0.0663	0.1658	0.2194	0.3776	0.1709

The following is the First-Level Fuzzy Comprehensive Evaluation:

WC1*RC1=[0.0904,0.1663,0.3129,0.2193,0.2110]

WC2*RC2=[0.0880,0.1046,0.1696,0.3163,0.3214]

WC3*RC3=[0.0934,0.1741,0.4000,0.2008,0.1317]

WC4*RC4=[0.0774,0.0961,0.2849,0.3325,0.2092]

WC5*RC5=[0.0761,0.1159,0.2295,0.3011,0.2775]

WC6*RC6=[0.0570,0.1173,0.2219,0.2806,0.3231]

WC7*RC7=[0.1114,0.2372,0.3146,0.1990,0.1378]

WC8*RC8=[0.0859,0.1344,0.1939,0.2551,0.3308]

WC9*RC9=[0.1011,0.1340,0.2875,0.2421,0.2352]

WC10*RC10=[0.0680,0.1454,0.2168,0.3070,0.2628]

WC11*RC11=[0.0702,0.1320,0.3068,0.2720,0.2189]

WC12*RC12=[0.0901,0.1293,0.2168,0.3461,0.2177]

WC13*RC13=[0.0689,0.1105,0.2619,0.3206,0.2381]

WC14*RC14=[0.0893,0.1352,0.3820,0.2283,0.1652]

Second-Level Fuzzy Comprehensive Evaluation

The fuzzy matrix (membership degree matrix) corresponding to B1 is obtained as: Table15

B1	The fuzzy	The fuzzy	The fuzzy	The fuzzy	The fuzzy
C1	0.0904	0.1663	0.3129	0.2193	0.2110
C2	0.0880	0.1046	0.1696	0.3163	0.3214

The fuzzy matrix (membership degree matrix) corresponding to B2 is obtained as: Table16

B2	The fuzzy	The fuzzy	The fuzzy	The fuzzy	The fuzzy
C3	0.0934	0.1741	0.4000	0.2008	0.1317
C4	0.0774	0.0961	0.2849	0.3325	0.2092

The fuzzy matrix (membership degree matrix) corresponding to B3 is obtained as: Table17

B3	The fuzzy	The fuzzy	The fuzzy	The fuzzy	The fuzzy
C5	0.0761	0.1159	0.2295	0.3011	0.2775

C6	0.0570	0.1173	0.2219	0.2806	0.3231
C7	0.1114	0.2372	0.3146	0.1990	0.1378

The fuzzy matrix (membership degree matrix) corresponding to B4 is obtained as: Table18

B4	The fuzzy	The fuzzy	The fuzzy	The fuzzy	The fuzzy
C8	0.0859	0.1344	0.1939	0.2551	0.3308
C9	0.1011	0.1340	0.2875	0.2421	0.2352
C10	0.0680	0.1454	0.2168	0.3070	0.2628

The fuzzy matrix (membership degree matrix) corresponding to B5 is obtained as: Table19

B5	The fuzzy	The fuzzy	The fuzzy	The fuzzy	The fuzzy
C11	0.0702	0.1320	0.3068	0.2720	0.2189
C12	0.0901	0.1293	0.2168	0.3461	0.2177

The fuzzy matrix (membership degree matrix) corresponding to B6 is obtained as: Table20

B6	The fuzzy	The fuzzy	The fuzzy	The fuzzy	The fuzzy
C13	0.0689	0.1105	0.2619	0.3206	0.2381
C14	0.0893	0.1352	0.3820	0.2283	0.1652

The following is the Second-Level Fuzzy Comprehensive Evaluation:

WB1*RB1=[0.0896,0.1458,0.2652,0.2517,0.2478]

WB2*RB2=[0.0880,0.1481,0.3616,0.2447,0.1575]

WB3*RB3=[0.0833,0.1539,0.2545,0.2653,0.2430]

WB4*RB4=[0.0852,0.1370,0.2230,0.2648,0.2899]

WB5*RB5=[0.0802,0.1306,0.2618,0.3091,0.2183]

WB6*RB6=[0.0757,0.1188,0.3019,0.2898,0.2138]

Third-Level Fuzzy Comprehensive Evaluation:

The fuzzy matrix (membership degree matrix) corresponding to U is obtained as: Table21

U	The fuzzy	The fuzzy	The fuzzy	The fuzzy	The fuzzy
B1	0.0896	0.1458	0.2652	0.2517	0.2478
B2	0.0880	0.1481	0.3616	0.2447	0.1575
B3	0.0833	0.1539	0.2545	0.2653	0.2430
B4	0.0852	0.1370	0.2230	0.2648	0.2899
B5	0.0802	0.1306	0.2618	0.3091	0.2183
B6	0.0757	0.1188	0.3019	0.2898	0.2138

The following is the Third-Level Fuzzy Comprehensive Evaluation:

X=WU*RU=[0.0848,0.1405,0.2752,0.2659,0.2335]

Let the grade score $Y=[1, 2, 3, 4, 5]^T$,

Therefore, the final evaluation score Z = X*Y = 3.4228 is obtained. From this, it can be inferred that the overall level of the sample is between medium and good.
